# Supplementary material for: Systematic exploration of Escherichia coli phage–host interactions with the BASEL phage collection
Source: PLoS Biol. 2021 Nov 16;19(11):e3001424. doi: 10.1371/journal.pbio.3001424 (PMC8594841; doi:10.1371/journal.pbio.3001424)
Supplement: S1 Table — The abbreviations in the selection column indicate the drug and its concentration that were used. Amp, ampicillin; Cam, chloramphenicol; Kan, kanamycin; Zeo, zeocin; 25/50/100 refer to 25 μg/ml, 50 μg/ml, and 100 μg/ml, respectively. The following mutants of the KEIO collection were used for qualitative top agar assays but are not included in the strain list because no phage showed any growth phenotype on them: ompW::kanR, phoE::kanR, flgG::kanR, fepA::kanR, hofQ::kanR, cirA::kanR, fhuE::kanR, fiu::kanR, ompN::kanR, pgaA::kanR, chiP::kanR, ompL::kanR, yddB::kanR, fecA::kanR, uidC::kanR, nanC::kanR, yfaZ::kanR, bglH::kanR, bcsC::kanR, cusC::kanR, gfcE::kanR, mdtP::kanR, ompG::kanR, ompX::kanR, yfeN::kanR, csgF::kanR, wza::kanR, flu::kanR, nmpC::kanR, eaeH::kanR, ydiY::kanR, yiaT::kanR, yaiO::kanR, mdtQ::kanR, pgaB::kanR, mipA::kanR, pldA::kanR, yzcX::kanR, ydeT::kanR, blc::kanR, gspD::kanR, yjgL::kanR. (DOCX) [file pbio.3001424.s001.docx]

# S1 Table. List of all bacterial strains used in this study

| **Strain** | **Genotype** | **relevant plasmid** | **Selection** | **Source/Description** |
| --- | --- | --- | --- | --- |
| AH-E02-148 | *Escherichia coli* K-12 MG1655: F^–^ λ^–^ *ilvG*^–^ *rfb-50 rph-1* | none | none | *E. coli* K-12 laboratory wildtype strain; obtained from the Coli Genetic Stock Center (CGSC #6300) via Prof. Urs Jenal |
| AH-E01-047 | *Escherichia coli* K-12 BW25113: F^-^ Δ(*araD-araB*)567, Δ*lacZ4787(::rrnB-3*), λ^-^, *rph-1*, Δ(*rhaD-rhaB*)568, *hsdR514* | none | none | our laboratory collection [1] |
| AH-E02-160 | *E. coli* K-12 MG1655 Δ*mrr-hsdRMS-mcrBC* | pWRG99 | Amp100 | this study |
| AH-E03-200 | *E. coli* K-12 MG1655 Δ*mrr-hsdRMS-mcrBC* Δ*mcrA* = ΔRM | none | none | this study; strain lacking all known restriction systems of *E. coli* K-12 |
| AH-E03-217 | *E. coli* K-12 MG1655 ΔRM | pBR322_ΔP*tet*  F(*pifA::zeoR*) | Amp50  Zeo50 | this study |
| AH-E06-427 | *E. coli* K-12 MG1655 ΔRM | pBR322_ΔP*tet*  pAH200e | Amp50  Kan25 | this study; pAH200e (F-plasmid tagged with kanamycin resistance at *tn1000* obtained from Prof. Christoph Dehio) |
| AH-E07-555 | *E. coli* K-12 W1872 | F | none | *E. coli* K-12 strain carrying a wildtype F-plasmid |
| AH-E03-233 | *E. coli* K-12 MG1655 ΔRM *waaC::kanR* | none | Kan25 | this study |
| AH-E03-235 | *E. coli* K-12 MG1655 ΔRM *waaG::kanR* | none | Kan25 | this study |
| AH-E03-243 | *E. coli* K-12 MG1655 ΔRM *wbbL(+)* | none | none | this study |
| AH-E04-321 | *E. coli* K-12 BW25113 *btuB::kanR* | none | Kan25 | this study |
| MBu-E01-044 | *E. coli* K-12 BW25113 *tolC::kanR* | none | Kan25 | this study |
| MBu-E01-021 | *E. coli* K-12 BW25113 *wecB::kanR* | none | Kan25 | obtained from Prof. Urs Jenal (KEIO collection [2]) |
| MBu-E01-007 | *E. coli* K-12 BW25113 *fhuA::kanR* | none | Kan25 | obtained from Prof. Urs Jenal (KEIO collection [2]) |
| AH-E05-396 | *E. coli* K-12 BW25113 *yncD::kanR* | none | Kan25 | obtained from Prof. Urs Jenal (KEIO collection [2]) |
| MBu-E01-018 | *E. coli* K-12 BW25113 *lamB::kanR* | none | Kan25 | obtained from Prof. Urs Jenal (KEIO collection [2]) |
| MBu-E01-015 | *E. coli* K-12 BW25113 *tsx::kanR* | none | Kan25 | obtained from Prof. Urs Jenal (KEIO collection [2]) |
| MBu-E01-016 | *E. coli* K-12 BW25113 *fadL::kanR* | none | Kan25 | obtained from Prof. Urs Jenal (KEIO collection [2]) |
| MBu-E01-011 | *E. coli* K-12 BW25113 *ompA::kanR* | none | Kan25 | obtained from Prof. Urs Jenal (KEIO collection [2]) |
| MBu-E01-012 | *E. coli* K-12 BW25113 *ompC::kanR* | none | Kan25 | obtained from Prof. Urs Jenal (KEIO collection [2]) |
| MBu-E01-013 | *E. coli* K-12 BW25113 *ompF::kanR* | none | Kan25 | obtained from Prof. Urs Jenal (KEIO collection [2]) |
| AH-E07-546 | *E. coli* K-12 BW25113 *lptD_*Δ(L394-V396)::Y | none | none | this study; spontaneous mutant resistant to LptD-targeting siphoviruses |
| AH-E07-545 | *E. coli* K-12 BW25113 *lptD_*Δ(Y658-Y678)::H | none | none | this study; spontaneous mutant resistant to LptD-targeting siphoviruses |
| AH-E01-044 | *E. coli* B REL606 | none | none | obtained from Dr. Jenna Gallie |
| AH-E03-168 | *E. coli* UTI89 | none | none | obtained from Prof. Urs Jenal |
| AH-E04-284 | *E. coli* CFT073 *rpoS(+)* | none | none | our laboratory collection [3] |
| AH-E06-481 | *E. coli* 55989 | none | none | our laboratory collection [3] |
| AH-E04-297 | *Salmonella enterica* subsp. *enterica* serovar Typhimurium 12023s (also known as ATCC 14028) | none | none | obtained from Prof. Dirk Bumann |
| AH-E06-438 | *S.* Typhimurium SL1344 | none | none | obtained from Prof. Mederic Diard |
| AH-E03-169 | *E. coli* K-12 EMG2 | none | none | most ancestral available *E. coli* K-12 strain; obtained from the Coli Genetic Stock Center (CGSC #4401) |
| AH-E01-053 | *E. coli* EB1484 (lysogen of phage P1 *clr100Km*) | P1 prophage | none | lysogen of a temperature-inducible P1 prophage tagged with kanamycin resistance; obtained from Prof. Kenneth Kreuzer |

# References (S1 Table)

1. Datsenko KA, Wanner BL. One-step inactivation of chromosomal genes in *Escherichia coli* K‑12 using PCR products. Proc Natl Acad Sci USA. 2000;97(12):6640-5. doi: 10.1073/pnas.120163297. PubMed PMID: 10829079; PubMed Central PMCID: PMC18686.

2. Baba T, Ara T, Hasegawa M, Takai Y, Okumura Y, Baba M, et al. Construction of *Escherichia coli* K-12 in-frame, single-gene knockout mutants: the Keio collection. Mol Syst Biol. 2006;2:2006 0008. doi: 10.1038/msb4100050. PubMed PMID: 16738554; PubMed Central PMCID: PMC1681482.

3. Fino C, Vestergaard M, Ingmer H, Pierrel F, Gerdes K, Harms A. PasT of *Escherichia coli* sustains antibiotic tolerance and aerobic respiration as a bacterial homolog of mitochondrial Coq10. Microbiologyopen. 2020;9(8):e1064. Epub 2020/06/20. doi: 10.1002/mbo3.1064. PubMed PMID: 32558363; PubMed Central PMCID: PMCPMC7424257.
