# Supplementary material for: Systematic exploration of Escherichia coli phage–host interactions with the BASEL phage collection
Source: PLoS Biol. 2021 Nov 16;19(11):e3001424. doi: 10.1371/journal.pbio.3001424 (PMC8594841; doi:10.1371/journal.pbio.3001424)
Supplement: S2 Table — (DOCX) [file pbio.3001424.s002.docx]

# S2 Table. List of all oligonucleotide primers used in this study

| ***Primer name*** | ***Sequence (5'-3')*** |
| --- | --- |
| prAH1815 | GCCGCTCCCGATGTGGTGTCGGGAGCGGTATTTTCTATAAAACTTACCGCGGAACTTCATTTAAATGGCG |
| prAH1816 | AGTAAGGGGTTATGGGCCGGATAAGGCGCAGCCGCATCCGGCCTGATATTGGTCCATATGAATATCCTCCTTAG |
| prAH1817 | ATGTGGTGTCGGGAGCGGTATTTTCTATAAAACTTACCGCAATATCAGGCCGGATGCGGCTGCGCCTTATCCGGCCCATA |
| prAH1818 | TATGGGCCGGATAAGGCGCAGCCGCATCCGGCCTGATATTGCGGTAAGTTTTATAGAAAATACCGCTCCCGACACCACAT |
| prAH1823 | TCAATAAAAGTAGTATTGTCGTGAAAAATTGATTAAAGATTAATATTATGGGTCCATATGAATATCCTCCTTAG |
| prAH1825 | ACGCCCGTTTCAATATTTAACACATGGAGAGATTACATGTTTTCGATGATGGAACTTCATTTAAATGGCG |
| prAH1826 | TAGTATTGTCGTGAAAAATTGATTAAAGATTAATATTATGATCATCGAAAACATGTAATCTCTCCATGTGTTAAATATTG |
| prAH1827 | CAATATTTAACACATGGAGAGATTACATGTTTTCGATGATCATAATATTAATCTTTAATCAATTTTTCACGACAATACTA |
| prAH1907 | GAAAGCCATCCAGTTTACTTTG |
| prAH1944 | GCTGGCGGAGGCCCTGACCGGGCTGCGCTGCCTGTACGGAGATCTGTAATTACAACTTTTTTTACTTCTTGTTCATTAG |
| prAH1945 | ATCTCCTTCATTTAGTAAGAAAAAGGCCGCTAAGCGGCCTTAATTTTTGGCTTTTGCTCACATGTTGGTC |
| prAH1948 | GGGAACGCGGCCGCACCTACATCTGTATTAACGAAGCG |
| prAH1949 | CTGCTTCTCGAGACACGGTGCCTGACTGC |
| prAH1950 | GGGTGACTCGAGATGAAGAATGGTTTTTATGCG |
| prAH1951 | GCATTTGCGGCCGCTTATTCTTGTTCTCTGGTCAAATTATAT |
| prAH1956 | GGGAGAATCGATAGAATCAGGTAGATGTTTTTCGG |
| prAH1957 | GCATTTGCGGCCGCTCAGGATTTTTTACGTGAGGC |
| prAH1981 | CACCTACATCTGTATTAACGAAGC |
| prAH1982 | GATAAGCTGTCAAACATGAGAATTC |
| prAH2009 | AATCAACAACCGTATCAGAATAGATACTTTCTTTAGGAATTTTTGTTTTACGTAATTTTTTTAAGGCAGTTATTG |
| prAH2010 | AAATTTCCTGTGCTTTCTGATTTTATTGTGTCATTTATGTTAGGGATTAACTTACTGTCCCTAGTGCTTGG |
| prAH2013 | GTGAAAAACTGATGAAATTCGAT |
| prAH2014 | GACATGAAGACTACATCAAAAAATTACT |
| prAH2015 | GTTATCACCAGAGCTTAATCGAC |
| prAH2016 | TATCTCTAAAATCATTGATGATTTCAG |
| prAH2019 | GTTTTTTGACCTCTGCAAAAG |
| prAH2020 | TGACCGCAACAAAAAATATC |
| prAH2184 | GAATTCTCATGTTTGACAGCTTATCACAGCTTAAAAACGAACTTGAAG |
| prAH2185 | CGCTTCGTTAATACAGATGTAGGTGCTTACTTCACCACTTCCATCAG |
| prAH2224 | CCCTTTGATATGTAACGGTGAAC |
| prAH2225 | GTTAATGTCATGATAATAATGGTTTCTTAGAC |
| prAH2266 | GGTATTTTCTCCTTACGCATCTG |
| prAH2319 | CAAGGCGATTAAGTTGGGTAA |
| prAH2343 | GGTTTCTTAGACGTCAGGTGG |
| prAH2344 | AGTGCCACCTGACGTCTAAGAAACCCATTACAAGAGTTTGCTGACAAG |
| prAH2345 | CACAGATGCGTAAGGAGAAAATACCCTATTTTTAACGACCTGAGCG |
| prAH2346 | AGTGCCACCTGACGTCTAAGAAACCTGTTAGAGTTGATACGGTTCCTG |
| prAH2347 | CACAGATGCGTAAGGAGAAAATACCTGGTGATGTGAATAAAGCGG |
| prAS046 | GAATTCTCATGTTTGACAGCTTATCTTAAATCCATTTTATGAAATCTTCC |
| prAS047 | CGCTTCGTTAATACAGATGTAGGTGACTAATGAGCCATCAGTATTTCC |
| prAS048 | GAATTCTCATGTTTGACAGCTTATCTCAATTGAGTATCGATTTTCGT |
| prAS049 | CGCTTCGTTAATACAGATGTAGGTGACAGCACAGTACTAAACCAATAGTG |
| prAS050 | GAATTCTCATGTTTGACAGCTTATCATTACTATGAGGTGAATGGCAAG |
| prAS051 | CGCTTCGTTAATACAGATGTAGGTGTCTGACAGTTTCCTTTGAGC |
| prEM0261 | GGCGTTACCCAACTTAATCGCCTTGGAAGAGAATCGATGTGAAAGTACT |
| prEM0262 | CACAGATGCGTAAGGAGAAAATACCAAATGGTCGCAAAACTCATAG |
| prEM0263 | GGCGTTACCCAACTTAATCGCCTTGATTGCTCATCAGATGTCCAG |
| prEM0264 | CACAGATGCGTAAGGAGAAAATACCACGTGGTTCAGAAGGTGTAG |
| prEM0265 | GGCGTTACCCAACTTAATCGCCTTGCTTAAGCATTGTTAATGTCCTGG |
| prEM0266 | CACAGATGCGTAAGGAGAAAATACCGTCGTCGTCATCAGTTACG |
| prEM0267 | GGCGTTACCCAACTTAATCGCCTTGCATCCCATCTAAGATATTAACCCT |
| prEM0268 | CACAGATGCGTAAGGAGAAAATACCAACAGCCAACTTGTGAAATG |
| prEM0269 | GGCGTTACCCAACTTAATCGCCTTGTTTTTGCTGCTTCACAATG |
| prEM0270 | CACAGATGCGTAAGGAGAAAATACCATCGTTAAAGTAAATGCCCG |
| prEM0271 | GGCGTTACCCAACTTAATCGCCTTGAAAAGCAATGACTCAGGAGATAG |
| prEM0272 | CACAGATGCGTAAGGAGAAAATACCTGCTATTACCACCAGATTTCC |
| prEM0273 | GGCGTTACCCAACTTAATCGCCTTGCGCAATCGATTACGTAAATG |
| prEM0274 | CACAGATGCGTAAGGAGAAAATACCCACGTTGTTAACAATCAGAAATG |
| prEM0275 | GGCGTTACCCAACTTAATCGCCTTGTGGATCGCGTTTCTTAGAT |
| prEM0276 | CACAGATGCGTAAGGAGAAAATACCTGCTATGCAGGTGACTTTATC |
| prEM0277 | GGCGTTACCCAACTTAATCGCCTTGTCTCTTCTGTAAATTGTCGCTG |
| prEM0278 | CACAGATGCGTAAGGAGAAAATACCTTCTACCAGACGAGAACTTAAGC |
| prEM0279 | GGCGTTACCCAACTTAATCGCCTTGTTCCCTTGCATTTACATTTTG |
| prEM0280 | CACAGATGCGTAAGGAGAAAATACCGCCCTTTGTTCGATATCAATC |
| prEM0281 | GGCGTTACCCAACTTAATCGCCTTGGAAATATGACGGTGTTCACAAA |
| prEM0282 | CACAGATGCGTAAGGAGAAAATACCACGTGAGATTGCTCTGGAAG |
| prEM0283 | GGCGTTACCCAACTTAATCGCCTTGGGCTATCTCCCACAATATAAAGG |
| prEM0284 | CACAGATGCGTAAGGAGAAAATACCCCATTTCAATTAACCGCACT |
| prEM0285 | GGCGTTACCCAACTTAATCGCCTTGTGTTACAAGAGGAAGCCTGAC |
| prEM0286 | CACAGATGCGTAAGGAGAAAATACCAGGATGTTAGCATGTTTTACCTTT |
| prEM0287 | GGCGTTACCCAACTTAATCGCCTTGGGAATGCACAATTATGATCG |
| prEM0288 | CACAGATGCGTAAGGAGAAAATACCTTAATTCAACCATCTAAACCACC |
| prEM0289 | TGCCTTCCTGATGATTATGT |
| prEM0290 | AAATCAGGTTCGACGATATG |
| prEM0291 | TATTAACCCTTCAGGAACGC |
| prEM0292 | GGTATCGGAATGATTCGTGTA |
| prEM0293 | ACAATCGTTAAAGTAAATGCCC |
| prEM0294 | CTTCACAATGCTTCGCTAAG |
| prMBu0003 | GGGTAAGGAATAACTGACGAAAG |
| prMBu0004 | GTAGAGTTAATATTGAGCAGATCCC |
| prMBu0005 | GCTTATTTCCATACGTGATTATCC |
| prMBu0006 | GATTATTCTGCATTTTTGGGG |
| prMBu0007 | CCAACTATTACTGATGAAAACGC |
| prMBu0008 | CCGTAAATTTTTATTGACAGAACTT |
| prMBu0011 | GCTTATCAGGCCTACGTTATTTC |
| prMBu0012 | GGCTCCTCTGTATACGAAATATTT |
| prMBu0015 | ACCTATACTCTCGCCACTGG |
| prMBu0016 | GCAAAACTCAGGAGGTAAGC |
| prMBu0021 | GTTTAAGCACCCCACAAAA |
| prMBu0022 | CCGTTGTAGGCCTGATAAGA |
| prMBu0025 | TAATATTGATGAAACCTGCGGCATCCTTCTTCTATTGTGGATGCTTTACAACCTGCAGTTCGAAGTTCC |
| prMBu0026 | CGTGTCGGTAATCGCATTGCGCGCATCGACATAATCATAACTCACAGTATGAGCTGCTTCGAAGTTCCTA |
| prMBU0027 | CTATTTGTGAGCTACGTCTGGA |
| prMBU0028 | AGTAATACCCCAGTCGAAGTCA |
| prMBU0071 | AATTGAGGCACATTAACGC |
| prMBU0072 | AGGATGGCTGGTCGAAAT |
| prMBu0075 | TACAGTTTGATCGCGCTAAATACTGCTTCACCACAAGGAATGCAAACCTGCAGTTCGAAGTTCC |
| prMBu0076 | TACGTTGCCTTACGTTCAGACGGGGCCGAAGCCCCGTCGTCGTCATGTAGGCTGGAGCTGCTTC |
| prMBu0078 | AAACCATTATTATCATGACATTAACTTCAAGAATACGGCTGGTC |
| prMBu0079 | CTGTTCACCGTTACATATCAAAGGGTCAATCATCTTATCGACTACCTTG |
