# Supplementary material for: Systematic exploration of Escherichia coli phage–host interactions with the BASEL phage collection
Source: PLoS Biol. 2021 Nov 16;19(11):e3001424. doi: 10.1371/journal.pbio.3001424 (PMC8594841; doi:10.1371/journal.pbio.3001424)
Supplement: S3 Table — The abbreviations in the selection column indicate the drug and its concentration that were used. Amp, ampicillin; Cam, chloramphenicol; Kan, kanamycin; Zeo, zeocin; 25/50/100 refer to 25 μg/ml, 50 μg/ml, and 100 μg/ml, respectively. (DOCX) [file pbio.3001424.s003.docx]

# S3 Table. List of all plasmids used in this study

| **name** | **Selection** | **Description** | **Source** |
| --- | --- | --- | --- |
| pWRG99 | Amp100 | lambda red recombineering plasmid with inducible I-SceI | our laboratory collection [1] |
| pWRG100 | Cam25 | template plasmid for the recombineering double-selectable cassette encoding chloramphenicol resistance and an I-SceI recognition site | our laboratory collection [1] |
| pJM05 | Kan25 | template plasmid for the recombineering double-selectable cassette encoding kanamycin resistance and *sacB* | our laboratory collection [2] |
| pUA139 | Kan25 | SC101 origin of replication, kanamycin resistance, and *gfpmut2* (originally to clone promoter-GFP fusions) | our laboratory collection [3] |
| pUA139_T7(*gp17*) | Kan25 | encoding part of the *gp17* tail fiber gene of phage T7 with flanking sequence | this study |
| pUA139_cat-sacB_v3 | Cam25, Kan25 | template plasmid for recombineering double-selectable cassettes encoding *sacB* and either chloramphenicol or kanamycin resistance | our laboratory collection |
| pPICZa | Zeo50 | *Pichia pastoris* expression vector with a zeocin resistance cassette | obtained from Prof. Urs Jenal |
| pAR280 | Amp50 | mini-R1 plasmid encoding the intact *E. coli* K‑12 *wbbL* open reading frame | obtained from Prof. Urs Jenal |
| pBR322_ΔP*tet* | Amp50 | variant of pBR322 in which the tetracycline resistance cassette and its promoter have been deleted; empty-vector control for immunity experiments | obtained from Prof. Călin Guet via Dr. David Thaler [4] |
| pAH186_SC101e | Amp50 | plasmid encoding ampicillin resistance and an SC101 low-copy origin of replication | our laboratory collection [5] |
| pAH213_EcoKI | Amp50 | pBR322 derivative expressing type I RM system EcoKI of *E. coli* K‑12 | this study |
| pAH213_EcoCFT_I | Amp50 | pAH186_SC101e derivative expressing type I RM system EcoCFT_I of *E. coli* CFT073 | this study |
| pEcoRI | Amp50 | pBR322 derivative expressing type II RM system EcoRI | obtained from Prof. Călin Guet [4] |
| pEcoRV | Amp50 | pBR322 derivative expressing type II RM system EcoRII | obtained from Prof. Călin Guet [4] |
| pAH213_EcoCFT_II | Amp50 | pBR322 derivative expressing type III RM system EcoCFT_II of *E. coli* CFT073 | this study |
| pAH213_EcoP1_I | Amp50 | pAH186_SC101e derivative expressing type I RM system EcoP1_I of *E. coli* phage P1 | this study |
| pAH213_RexAB | Amp50 | pBR322 derivative expressing the RexAB Abi system of *E. coli* phage lambda | this study |
| pAH200e | Kan25 | F-plasmid in which the *tn1000* locus was replaced with a kanamycin resistance cassette by recombineering | obtained from Prof. Christoph Dehio |
| pAH213_Fun/Z | Amp50 | pBR322 derivative expressing the Fun/Z Abi system of *E. coli* phage P2 | this study |
| pAH213_Old | Amp50 | pBR322 derivative expressing the Old Abi system of *E. coli* phage P2 | this study |
| pAH213_Tin | Amp50 | pBR322 derivative expressing the Tin Abi system of *E. coli* phage P2 | this study |
| pAH186_SC101_btuB | Amp50 | pAH186_SC101e derivative expressing *btuB* of *E. coli* K-12 BW25113 | this study |
| pAH186_SC101_tolC | Amp50 | pAH186_SC101e derivative expressing *tolC* of *E. coli* K-12 BW25113*3* | this study |
| pAH186_SC101_fhuA | Amp50 | pAH186_SC101e derivative expressing *fhuA* of *E. coli* K-12 BW25113 | this study |
| pAH186_SC101_yncD | Amp50 | pAH186_SC101e derivative expressing *yncD* of *E. coli* K-12 BW25113 | this study |
| pAH186_SC101_lamB | Amp50 | pAH186_SC101e derivative expressing *lamB* of *E. coli* K-12 BW25113 | this study |
| pAH186_SC101_tsx | Amp50 | pAH186_SC101e derivative expressing *tsx* of *E. coli* K-12 BW25113 | this study |
| pAH186_SC101_ompA | Amp50 | pAH186_SC101e derivative expressing *ompA* of *E. coli* K-12 BW25113 | this study |
| pAH186_SC101_ompC | Amp50 | pAH186_SC101e derivative expressing *ompC* of *E. coli* K-12 BW25113 | this study |
| pAH186_SC101_waaC | Amp50 | pAH186_SC101e derivative expressing *waaC* of *E. coli* K-12 BW25113 | this study |
| pAH186_SC101_waaG | Amp50 | pAH186_SC101e derivative expressing *waaG* of *E. coli* K-12 BW25113 | this study |
| pAH186_SC101_wecB | Amp50 | pAH186_SC101e derivative expressing *wecB* of *E. coli* K-12 BW25113 | this study |
| pAH186_SC101_fadL | Amp50 | pAH186_SC101e derivative expressing *fadL* of *E. coli* K-12 BW25113 | this study |
| pAH186_SC101_ompF | Amp50 | pAH186_SC101e derivative expressing *ompF* of *E. coli* K-12 BW25113 | this study |
| pAH186_SC101_lptD | Amp50 | pAH186_SC101e derivative expressing *lptD* of *E. coli* K-12 BW25113 | this study |

# References (S3 Table)

1. Blank K, Hensel M, Gerlach RG. Rapid and highly efficient method for scarless mutagenesis within the *Salmonella enterica* chromosome. PloS one. 2011;6(1):e15763. doi: 10.1371/journal.pone.0015763. PubMed PMID: 21264289; PubMed Central PMCID: PMCPMC3021506.

2. MacKichan JK, Gerns HL, Chen YT, Zhang P, Koehler JE. A SacB mutagenesis strategy reveals that the *Bartonella quintana* variably expressed outer membrane proteins are required for bloodstream infection of the host. Infect Immun. 2008;76(2):788-95. doi: 10.1128/IAI.01174-07. PubMed PMID: 18070893; PubMed Central PMCID: PMC2223462.

3. Zaslaver A, Bren A, Ronen M, Itzkovitz S, Kikoin I, Shavit S, et al. A comprehensive library of fluorescent transcriptional reporters for *Escherichia coli*. Nat Methods. 2006;3(8):623-8. doi: 10.1038/nmeth895. PubMed PMID: 16862137.

4. Pleska M, Qian L, Okura R, Bergmiller T, Wakamoto Y, Kussell E, et al. Bacterial Autoimmunity Due to a Restriction-Modification System. Curr Biol. 2016;26(3):404-9. Epub 2016/01/26. doi: 10.1016/j.cub.2015.12.041. PubMed PMID: 26804559.

5. Fino C, Vestergaard M, Ingmer H, Pierrel F, Gerdes K, Harms A. PasT of *Escherichia coli* sustains antibiotic tolerance and aerobic respiration as a bacterial homolog of mitochondrial Coq10. Microbiologyopen. 2020;9(8):e1064. Epub 2020/06/20. doi: 10.1002/mbo3.1064. PubMed PMID: 32558363; PubMed Central PMCID: PMCPMC7424257.
