# Supplementary material for: Systematic exploration of Escherichia coli phage–host interactions with the BASEL phage collection
Source: PLoS Biol. 2021 Nov 16;19(11):e3001424. doi: 10.1371/journal.pbio.3001424 (PMC8594841; doi:10.1371/journal.pbio.3001424)
Supplement: S2 Text — BASEL, BActeriophage SElection for your Laboratory. (DOCX) [file pbio.3001424.s009.docx]

# S2 Text. Different considerations regarding the composition of the BASEL collection and the phenotypic analyses to characterize it

## Considerations regarding the composition of the BASEL collection

### Lack of O-antigen expression by *E. coli* K-12 ΔRM and exclusion of tailless phages

The choice of isolation host is known to be “perhaps the most critical part of the isolation process” because it pre-determines the properties and range of phages that can be sampled [1]. We therefore specifically designed a highly phage-sensitive host strain, *Escherichia coli* K-12 ΔRM, to eliminate as many biases in the isolation process as possible (see *Materials and Methods* as well as *Composition of the BASEL collection* in the main text).

However, one important feature of *E. coli* K-12 ΔRM is that it displays the rough LPS of all K12‑lineage laboratory strains and lacks the O-antigen chains that are otherwise fully covering the cell surface of natural enterobacterial isolates (Figs 2A and 11) [2]. On one hand, the absence of this formidable barrier enabled the isolation of a huge diversity of phages from all families that are, with very few exceptions, largely unable to infect *E. coli* K-12 with restored O16-type O‑antigen expression (Figs 2A, 3, and 5-10). However, using a strain with rough LPS as bait implicitly excluded the isolation of any phages for which the O-antigen is not only an optional primary receptor but an essential part of the host recognition. Two common examples for such phages are iconic *Salmonella* phage P22 and *Gamaleyavirus* G7C, a relative of N4 (see Fig 9E), that both bind to very specific types of O‑antigen and then target the glycan chain by enzymatic activities of their tailspikes to generate directional movement towards the cell surface [3, 4]. It is clear that this kind of phages are not highly abundant among *E. coli* phages because, e.g., a study that had isolated fifty phages primarily using strains with smooth LPS found 1) mostly the same taxonomic groups as those in the BASEL collection and reported that 2) many phages isolated on smooth hosts can also infect *E. coli* K-12 [5]. However, in order to clarify quantitatively how far the exclusion of O‑antigen-dependent phages affects the composition of the BASEL collection, we determined the proportion of O16-dependent phages among all phages able to infect *E. coli* K‑12 with restored O16-type O-antigen (S6 Fig). Across sewage inflow of four different treatment facilities in Switzerland, the proportion of O16-dependent phages was consistently very low (only few percent of the isolates; S6 Fig). Given this low abundance of O-antigen specialists and that most realistic applications of the BASEL collection will be based on infecting *E. coli* K-12 strains with rough LPS, we do not feel that the absence of these O-antigen-dependent phages compromises the usefulness of our work. However, we imagine that a future study could generate a dedicated, optional expansion of the BASEL collection containing a diverse and representative selection of these phages that are specialized in the O16-type O‑antigen of *E. coli* K‑12. Similar to these “O-antigen specialists”, we also excluded all tailless phages like *Microviridae* and *Inoviridae* from the BASEL collection because their biology, evolution, and host interactions are so different from the lytic *Caudovirales* that we feel their investigation deserves to be covered separately and is beyond the scope of this study [6, 7].

### Limitations due to the finite size of the BASEL collection

Any bacteriophage collection of finite size is inherently unable to include all rare phage groups and cannot comprehensively cover all genera of highly diverse and abundant families such as the *Drexlerviridae* (Fig 3D). Despite this shortcoming, we feel that the BASEL collection provides a reasonably complete overview of *E. coli* phage diversity because, e.g., all common and almost all previously described protein receptors of all included phage groups are covered (Figs 3‑10). Increasing the number of phage isolates in the BASEL collection might therefore not greatly increase its biological diversity but could jeopardize its usefulness by making the handling more complicated. It also does not appear that sampling most phages from sewage plant inflow is a major limitation, because we did not observe any difference in the phages sampled from sewage and, e.g., river water (S5 Table). Previous work had sampled overall similar sets of phage groups no matter if the phages came from sewage or from infant guts [5, 8-13], possibly because many *E. coli* phages in the environment are directly or indirectly derived from fecal contaminations.

### Limitations due to possible unrecognized mutations in the genomes of *E. coli* K-12 strains

Another limitation of our isolation host strain *E. coli* K-12 ΔRM is that it might introduce additional biases beyond its rough LPS phenotype. As an example, laboratory adaptation might have inactivated also other possible phage receptors on the cell surface. A relevant example for the possible consequences of such a bias is the observation that phage T5 of the T phages is a highly unusual member of the *Markadamsvirinae* subfamily of *Demerecviridae* because it uses the FhuA protein as its terminal receptor (Fig 6) [14]. This peculiar feature of the T phages is easily explained with the *btuB* loss-of-function mutation of many early *E. coli* B strains that were used to sample the T phages but later reverted back to functional BtuB expression, e.g., in the lineage leading to *E. coli* B REL606 that is sensitive to all tested *Markadamsvirinae* (Fig 12D) [14, 15]. Similarly, the T phages do not contain any *Vequintavirinae* phage despite the abundance of this group, and we indeed find that none of our twelve *Vequintavirinae sensu stricto* can lyse *E. coli* B REL606 (Fig 12F). Since this defect is probably due to impaired adsorption, we speculate that the truncated *E. coli* B LPS core and / or changes of the ECA or any other primary receptor might be responsible for this surprising phenotype. Though we cannot formally exclude hidden biases of our phage sampling due to possible mutations of the *E. coli* K-12 genomic backbone, the overall high similarity of taxonomic groups sampled in our study and previous work using different host strains (like the study by Korf et al. [5]) indicates that these possible biases cannot be severe.

### Limitations due to remaining immunity systems in *E. coli* K-12 ΔRM

Another possible factor biasing the diversity of isolated bacteriophages could be remaining immunity systems in the sampling strain *E. coli* K-12 ΔRM. While this strain lacks all known restriction systems of *E. coli* K-12 as well as the RexAB and PifA Abi systems (S1 Text), several systems remain that either have been poorly studied or are supposed to have only a narrow target range [16]. As an example, the cryptic prophage *e14* of *E. coli* K-12 encodes the Lit Abi system that cleaves the elongation factor Tu (EF‑Tu) when sensing the major capsid protein of *Tevenvirinae* [16, 17]. It is intuitive that the presence of *lit* in *E. coli* K-12 ΔRM might somehow bias the range of *Tevenvirinae* phages that we are sampling. However, although we confirmed by whole-genome sequencing that both the host as well as the T4 phage should carry alleles of *lit* and the major capsid protein gene triggering abortive infection [17], we observe robust plaque formation of T4 on our *E. coli* K-12 strains (S4A and S5A Figs). Similarly, recent work growing phage T4 on *E. coli* K‑12 hosts never reported any problems with abortive infection [5, 18, 19]. We are therefore skeptical if abortive infection by *lit* can significantly affect the isolation of bacteriophages with *E. coli* K-12 ΔRM. Besides Lit, the RnlAB type II toxin-antitoxin system aborts the growth of T4 by activation of the RnlA RNase toxin if the phage lacks the *dmd* antitoxin gene [16, 20]. However, given the broad conservation of *dmd* among *Tevenvirinae* and the absence of any reports that RnlAB could target other phages, we do not think that this system has a significant impact on our bacteriophage isolation experiments. Like for Lit and RnlAB, we see no evidence that the other proposed immunity systems of *E. coli* K-12 had relevant impact on the diversity of sampled phages. Notably, these *bona fide* immunity systems have often only been preliminarily characterized or, in case of DicB, were only shown to work when ectopically expressed [21].

## Considerations regarding the phenotyping of bacterial immunity systems

### Caveats due to the ectopic expression of bacterial immunity systems from plasmids

Our phage phenotyping experiments with diverse immunity systems were performed as quantitative top agar assays to determine the efficiency of plating (EOP) of each phage on a host carrying a given immunity system to generate robust, quantitative data (see *Materials and Methods*). However, there are a few technical caveats associated with our approach that need to be considered for a comprehensive interpretation of our results. Most importantly, for the phenotyping in the *E. coli* K-12 ΔRM host we cloned the different immunity systems onto plasmids which, though most were cloned with their native promoter, might affect their functionality by change of copy number (see *Materials and Methods* as well as S3 and S4 Tables). As an example, it was shown that phage T4 wildtype is resistant to RexAB but becomes sensitive when this immunity system is overexpressed from a multicopy plasmid [22]. However, the ColE1 and SC101 origins of replications used in this study have moderate to low copy numbers (of around 40 and 3-4, respectively [23]) and, e.g., our *rexAB* construct with ColE1 origin of replication has no detectable effect on the growth of phage T4 or any relative (Fig 7E), while an *rIIAB* mutant of this phage was sensitive as described previously (S5A and S5B Figs) [16]. The use of two different origins of replication is a consequence of our initial choice to clone immunity systems onto plasmids isogenic to the EcoRI and EcoRV plasmids of Pleška, Qian et al. [24] which later resulted in problems with toxicity for some constructs. These were then cloned into the lower-copy plasmid with SC101 origin of replication instead (S4 Table). Though expression levels might be different between these backbones as already evidenced from the difference in toxicity, we do not feel that this compromises our approach considerably. As an example, the two type III RM systems EcoP1_I and EcoCFT_II have a similar recognition sequence (Fig 2B) that is similarly abundant in the diverse phage genomes (S5 Table), but EcoP1_I was cloned with an SC101 origin of replication while EcoCFT_II was cloned with a ColE1 origin of replication. However, the phenotyping data do not show EcoCFT_II would be more potent compared to EcoP1_I (Figs 3 and 5-10) – sometimes one or the other has the stronger effect on EOP, sometimes they are similar.

### Representation of results for phages lacking recognition sites of restriction-modification systems

Another more transparent caveat is that the graphs displaying the EOP of different phages on hosts with restriction-modification (RM) systems are not directly comparable quantitatively. The reason is that, due to differences in genome size, GC content, and evolution towards restriction site avoidance, the different genomes have vastly different numbers of recognition sites for a given RM system [25] (listed in S5 Table). In extreme cases such as for type II RM systems and many tested podoviruses, recognition sites can be completely absent (S5 Table). One might argue that displaying an EOP (of ca. 1) for phage / RM interactions in the absence of recognition sites is not useful because – just biochemically – a cleavage of the phage chromosome was barely possible. However, given that the number of these sites is strongly under selection and part of the phages’ strategy to counter their host’s defenses, we find it more appropriate to show all data and highlight for the reader when no recognition sites were present.

### Effects of multiplicity of infection (MOI)

One dimension of bacterium-phage interactions that our work did not explicitly study was the question of whether changes in multiplicity of infection (MOI), i.e., the local number of virions per host cell, affect the potency of different immunity systems. Such phenomena had been described previously, e.g., for immunity mediated by CRISPR-Cas that can sometimes not be overcome by anti-CRISPR proteins if expressed from a single phage genome but, due to gene dosage effects, if a cell is infected by multiple virions [26]. Notably, our EOP assays always tested eight tenfold dilutions of each phage stock per host strain (see *Materials and Methods*) and would therefore have revealed strong MOI effects as an abrupt change in phage resistance / sensitivity from one dilution to the next. However, we never observed such a phenomenon. It could be that our practical choice of testing (most) bacterial immunity systems cloned onto plasmids (see above) has masked such MOI effects because a higher copy number of immunity systems would intuitively protect much stronger against phage gene dosage effects. We therefore look forward to future studies that might use the BASEL phages or other suitable models to study how the MOI affects the sensitivity / resistance of the various phage groups to the diverse immunity systems.

## Considerations regarding the phenotyping of knockout mutants with pleiotropic outer membrane phenotypes

In our study we used *waaC* and *waaG* knockout mutants to probe the dependency of bacteriophages on an intact *E. coli* K-12 LPS core (Fig 2A). The truncated nature of the LPS core expressed by these strains is well defined and understood [27]. However, the altered mechanical properties of the cell envelope and changes in the outer membrane proteome of *E. coli* K-12 mutants with truncated LPS core might also affect phage infectivity indirectly [28]. Nevertheless, we see no evidence that these indirect phenomena could have strongly affected our results. A wide diversity of phages ranging from T5-like siphoviruses of the *Markadamsvirinae* to podoviruses of the *Enquatrovirus* genus that use a variety of different receptors are not detectably affected by even the deepest viable core LPS truncation of the *waaC* mutant (Figs 6C-E and 9F). Other groups of phages like *Tevenvirinae* or small siphoviruses (*Drexlerviridae*, *Dhillonvirus*, *Nonagvirus*, and *Seuratvirus*) do show a marked heterogeneity in their sensitivity to LPS truncations, but these don’t correlate in any way with, e.g., the dependency on certain outer membrane proteins as receptors whose expression could be altered (Figs 3C-E, 5C-F, and 7C-E). Instead, we found a satisfactory explanation for the divergent LPS core dependency of T-even phages by uncovering that the members of this group each encode one of two distinct variants of short tail fibers that apparently target different parts of the LPS core (Fig 7E as well as S4B and S4C Figs). We find it highly likely that similar, direct effects are also at play for the small siphoviruses rather than that these would be divergently affected by ill-defined indirect consequences of LPS truncations.

Another potential confounding effect of LPS truncations is the activation of the Rcs system by cell surface stress which can result in a strong induction of colanic acid secretion [29] and, consequently, broad bacteriophage resistance [19, 30]. However, we did neither detect broad, unspecific phage resistance of the *waaC* and *waaG* mutants (Figs 3 and 5-10) nor any remarkable mucoidy that is characteristically associated with colanic acid overproduction [30]. Consistently, a dedicated study showed that under our standard laboratory conditions (LB agar plates, 37°C) only a *waaF* mutant but not the *waaC* or *waaG* mutants of *E. coli* K-12 produce colanic acid [31]. We therefore see no evidence that our results with the *waaC* and *waaG* mutants could be affected by colanic acid secretion induced by membrane stress responses.

Besides the *waaC* and *waaG* knockouts, the *tolC* knockout (lacking the major efflux pump outer membrane channel of *E. coli*) displays an increased outer membrane permeability reminiscent of (yet different from) deep-rough LPS core mutants [32]. Since a *tolC* knockout also showed resistance to a supposedly LPS-targeting bacteriophage, it was suggested that functional TolC might be important for an intact and correctly modified LPS core of *E. coli* K-12 [33]. However, we see no evidence that such a potential LPS core defect of the *tolC* knockout has affected our results. Only one tested phage, DanielBernoulli (Bas08), showed impaired infectivity on the *tolC* knockout mutant. This phage belongs to the *Tlsvirus* genus of *Drexlerviridae* that also includes other phages known to target TolC like LL5 and TLS which also share an orthologous *bona fide* receptor-binding protein (Figs 3D and 4B) [34, 35]. Unlike DanielBernoulli (Bas08), any possible LPS core defects of the *tolC* knockout did not considerably affect the infectivity of any other tested phage as can, e.g., be seen in S9 Fig where the absence of *tolC* only and specifically affects DanielBernoulli but none of the other phages spotted onto the same top agar. Notably, some of these other phages are exquisitely sensitive to any tested LPS core truncation like EmilieFrey (Bas61; see Fig 8D and the uncomplemented *waaC* or *waaG* knockouts in S11 and S12 Figs). This suggests that possible defects caused by the *tolC* knockout in the LPS core did not affect infection by any phage of the BASEL collection.

## Potential and limitations of the BASEL collection phages as new model organisms

We highlight already early in our manuscript (subsection *Composition of the BASEL collection* of the *Results* section) as well as towards the end (subsection *The BASEL collection [...]* of the *Discussion* section) that the BASEL collection roughly represents the diversity of common groups of *E. coli* phages. Together with our systematic characterization, we see these phages therefore as model organisms not unlike their *E. coli* K-12 host or, by extension, *Caenorhabditis elegans* worms, zebrafish, or mice. As we argue throughout the main manuscript with view to the BASEL collection, such model organisms are invaluable for their potential to unravel fundamental biological and evolutionary concepts [36]. However, a clear caveat in particular for the highly diverse world of bacteriophages is that these fundamental discoveries need to be tested in other systems as well to probe if or how far they can be generalized. We therefore envision future studies exploring, e.g., whether the taxonomic distribution of common phages infecting other Gram-negative or Gram-positive hosts would also fit the “royal family model” as we find for *E. coli* phages and as it was originally described for marine communities [37].

Similarly, the use of standard culture conditions (LB medium, 37°C, regular aeration) like for most bacteriophage work of the last decades was an obvious choice to generate the systematic data presented in this manuscript (see *Materials and Methods*). However, these conditions mimic only a small part of the diverse environments and physiological conditions that add up to the complex habitats of *E. coli* and its phages (if at all). An interesting application of the BASEL collection phages could therefore be to test how other, possibly physiologically more relevant conditions affect phage host range or the sensitivity / resistance of phages to bacterial immunity systems. These results might help to select phages, e.g., for therapeutic applications for which the insufficiency of standard laboratory conditions to determine the effective host range of bacteriophages inside patients has repeatedly been highlighted [1].

# References (S2 Text)

1. Hyman P. Phages for Phage Therapy: Isolation, Characterization, and Host Range Breadth. Pharmaceuticals (Basel). 2019;12(1). Epub 2019/03/14. doi: 10.3390/ph12010035. PubMed PMID: 30862020; PubMed Central PMCID: PMCPMC6469166.

2. Liu D, Reeves PR. *Escherichia* coli K12 regains its O antigen. Microbiology. 1994;140 ( Pt 1):49-57. doi: 10.1099/13500872-140-1-49. PubMed PMID: 7512872.

3. Broeker NK, Barbirz S. Not a barrier but a key: How bacteriophages exploit host's O-antigen as an essential receptor to initiate infection. Mol Microbiol. 2017;105(3):353-7. Epub 2017/06/16. doi: 10.1111/mmi.13729. PubMed PMID: 28618013.

4. Prokhorov NS, Riccio C, Zdorovenko EL, Shneider MM, Browning C, Knirel YA, et al. Function of bacteriophage G7C esterase tailspike in host cell adsorption. Mol Microbiol. 2017;105(3):385-98. Epub 2017/05/18. doi: 10.1111/mmi.13710. PubMed PMID: 28513100.

5. Korf IHE, Meier-Kolthoff JP, Adriaenssens EM, Kropinski AM, Nimtz M, Rohde M, et al. Still Something to Discover: Novel Insights into *Escherichia coli* Phage Diversity and Taxonomy. Viruses. 2019;11(5). doi: 10.3390/v11050454. PubMed PMID: 31109012; PubMed Central PMCID: PMCPMC6563267.

6. Hay ID, Lithgow T. Filamentous phages: masters of a microbial sharing economy. EMBO Rep. 2019;20(6). Epub 2019/04/07. doi: 10.15252/embr.201847427. PubMed PMID: 30952693; PubMed Central PMCID: PMCPMC6549030.

7. Doore SM, Fane BA. The microviridae: Diversity, assembly, and experimental evolution. Virology. 2016;491:45-55. Epub 2016/02/14. doi: 10.1016/j.virol.2016.01.020. PubMed PMID: 26874016.

8. Olsen NS, Forero-Junco L, Kot W, Hansen LH. Exploring the Remarkable Diversity of Culturable *Escherichia coli* Phages in the Danish Wastewater Environment. Viruses. 2020;12(9). Epub 2020/09/10. doi: 10.3390/v12090986. PubMed PMID: 32899836; PubMed Central PMCID: PMCPMC7552041.

9. Mathieu A, Dion M, Deng L, Tremblay D, Moncaut E, Shah SA, et al. Virulent coliphages in 1-year-old children fecal samples are fewer, but more infectious than temperate coliphages. Nat Commun. 2020;11(1):378. Epub 2020/01/19. doi: 10.1038/s41467-019-14042-z. PubMed PMID: 31953385; PubMed Central PMCID: PMCPMC6969025.

10. Sørensen PE, Van Den Broeck W, Kiil K, Jasinskyte D, Moodley A, Garmyn A, et al. New insights into the biodiversity of coliphages in the intestine of poultry. Sci Rep. 2020;10(1):15220. Epub 2020/09/18. doi: 10.1038/s41598-020-72177-2. PubMed PMID: 32939020; PubMed Central PMCID: PMCPMC7494930.

11. Michniewski S, Redgwell T, Grigonyte A, Rihtman B, Aguilo-Ferretjans M, Christie-Oleza J, et al. Riding the wave of genomics to investigate aquatic coliphage diversity and activity. Environ Microbiol. 2019;21(6):2112-28. Epub 2019/03/19. doi: 10.1111/1462-2920.14590. PubMed PMID: 30884081; PubMed Central PMCID: PMCPMC6563131.

12. Smith R, O'Hara M, Hobman JL, Millard AD. Draft Genome Sequences of 14 *Escherichia coli* Phages Isolated from Cattle Slurry. Genome Announc. 2015;3(6). Epub 2016/01/02. doi: 10.1128/genomeA.01364-15. PubMed PMID: 26722010; PubMed Central PMCID: PMCPMC4698387.

13. Pacifico C, Hilbert M, Sofka D, Dinhopl N, Pap IJ, Aspock C, et al. Natural Occurrence of *Escherichia coli*-Infecting Bacteriophages in Clinical Samples. Front Microbiol. 2019;10:2484. Epub 2019/11/19. doi: 10.3389/fmicb.2019.02484. PubMed PMID: 31736918; PubMed Central PMCID: PMCPMC6834657.

14. Demerec M, Fano U. Bacteriophage-Resistant Mutants in *Escherichia Coli*. Genetics. 1945;30(2):119-36. Epub 1945/03/01. PubMed PMID: 17247150; PubMed Central PMCID: PMCPMC1209279.

15. Studier FW, Daegelen P, Lenski RE, Maslov S, Kim JF. Understanding the differences between genome sequences of *Escherichia coli* B strains REL606 and BL21(DE3) and comparison of the *E. coli* B and K-12 genomes. J Mol Biol. 2009;394(4):653-80. doi: 10.1016/j.jmb.2009.09.021. PubMed PMID: 19765592.

16. Lopatina A, Tal N, Sorek R. Abortive Infection: Bacterial Suicide as an Antiviral Immune Strategy. Annu Rev Virol. 2020;7(1):371-84. Epub 2020/06/20. doi: 10.1146/annurev-virology-011620-040628. PubMed PMID: 32559405.

17. Yu YT, Snyder L. Translation elongation factor Tu cleaved by a phage-exclusion system. Proc Natl Acad Sci U S A. 1994;91(2):802-6. Epub 1994/01/18. doi: 10.1073/pnas.91.2.802. PubMed PMID: 8290603; PubMed Central PMCID: PMCPMC43037.

18. Trojet SN, Caumont-Sarcos A, Perrody E, Comeau AM, Krisch HM. The gp38 adhesins of the T4 superfamily: a complex modular determinant of the phage's host specificity. Genome Biol Evol. 2011;3:674-86. Epub 2011/07/13. doi: 10.1093/gbe/evr059. PubMed PMID: 21746838; PubMed Central PMCID: PMCPMC3157838.

19. Mutalik VK, Adler BA, Rishi HS, Piya D, Zhong C, Koskella B, et al. High-throughput mapping of the phage resistance landscape in *E. coli*. PLoS Biol. 2020;18(10):e3000877. Epub 2020/10/14. doi: 10.1371/journal.pbio.3000877. PubMed PMID: 33048924; PubMed Central PMCID: PMCPMC7553319 following competing interests: VKM, AMD, and APA consult for and hold equity in Felix Biotechnology, Inc.

20. Koga M, Otsuka Y, Lemire S, Yonesaki T. *Escherichia coli* *rnlA* and *rnlB* compose a novel toxin-antitoxin system. Genetics. 2011;187(1):123-30. Epub 2010/10/29. doi: 10.1534/genetics.110.121798. PubMed PMID: 20980243; PubMed Central PMCID: PMCPmc3018318.

21. Ragunathan PT, Vanderpool CK. Cryptic-Prophage-Encoded Small Protein DicB Protects *Escherichia coli* from Phage Infection by Inhibiting Inner Membrane Receptor Proteins. J Bacteriol. 2019;201(23). Epub 2019/09/19. doi: 10.1128/JB.00475-19. PubMed PMID: 31527115; PubMed Central PMCID: PMCPMC6832061.

22. Shinedling S, Parma D, Gold L. Wild-type bacteriophage T4 is restricted by the lambda *rex* genes. J Virol. 1987;61(12):3790-4. Epub 1987/12/01. doi: 10.1128/JVI.61.12.3790-3794.1987. PubMed PMID: 2960831; PubMed Central PMCID: PMCPMC255994.

23. Jahn M, Vorpahl C, Hubschmann T, Harms H, Muller S. Copy number variability of expression plasmids determined by cell sorting and Droplet Digital PCR. Microb Cell Fact. 2016;15(1):211. doi: 10.1186/s12934-016-0610-8. PubMed PMID: 27993152; PubMed Central PMCID: PMCPMC5168713.

24. Pleska M, Qian L, Okura R, Bergmiller T, Wakamoto Y, Kussell E, et al. Bacterial Autoimmunity Due to a Restriction-Modification System. Curr Biol. 2016;26(3):404-9. Epub 2016/01/26. doi: 10.1016/j.cub.2015.12.041. PubMed PMID: 26804559.

25. Rusinov IS, Ershova AS, Karyagina AS, Spirin SA, Alexeevski AV. Avoidance of recognition sites of restriction-modification systems is a widespread but not universal anti-restriction strategy of prokaryotic viruses. BMC Genomics. 2018;19(1):885. Epub 2018/12/12. doi: 10.1186/s12864-018-5324-3. PubMed PMID: 30526500; PubMed Central PMCID: PMCPMC6286503.

26. Borges AL, Zhang JY, Rollins MF, Osuna BA, Wiedenheft B, Bondy-Denomy J. Bacteriophage Cooperation Suppresses CRISPR-Cas3 and Cas9 Immunity. Cell. 2018;174(4):917-25 e10. Epub 2018/07/24. doi: 10.1016/j.cell.2018.06.013. PubMed PMID: 30033364; PubMed Central PMCID: PMCPMC6086726.

27. Bertani B, Ruiz N. Function and Biogenesis of Lipopolysaccharides. EcoSal Plus. 2018;8(1). Epub 2018/08/02. doi: 10.1128/ecosalplus.ESP-0001-2018. PubMed PMID: 30066669; PubMed Central PMCID: PMCPMC6091223.

28. Pagnout C, Sohm B, Razafitianamaharavo A, Caillet C, Offroy M, Leduc M, et al. Pleiotropic effects of *rfa*-gene mutations on *Escherichia coli* envelope properties. Sci Rep. 2019;9(1):9696. Epub 2019/07/06. doi: 10.1038/s41598-019-46100-3. PubMed PMID: 31273247; PubMed Central PMCID: PMCPMC6609704.

29. Wall E, Majdalani N, Gottesman S. The Complex Rcs Regulatory Cascade. Annu Rev Microbiol. 2018;72:111-39. Epub 2018/06/14. doi: 10.1146/annurev-micro-090817-062640. PubMed PMID: 29897834.

30. Chaudhry W, Lee E, Worthy A, Weiss Z, Grabowicz M, Vega N, et al. Mucoidy, a general mechanism for maintaining lytic phage in populations of bacteria. FEMS Microbiol Ecol. 2020;96(10). Epub 2020/08/28. doi: 10.1093/femsec/fiaa162. PubMed PMID: 32845324; PubMed Central PMCID: PMCPMC7532286.

31. Ren G, Wang Z, Li Y, Hu X, Wang X. Effects of Lipopolysaccharide Core Sugar Deficiency on Colanic Acid Biosynthesis in *Escherichia coli*. J Bacteriol. 2016;198(11):1576-84. Epub 2016/03/24. doi: 10.1128/JB.00094-16. PubMed PMID: 27002133; PubMed Central PMCID: PMCPMC4959291.

32. Fralick JA. Evidence that TolC is required for functioning of the Mar/AcrAB efflux pump of *Escherichia coli*. J Bacteriol. 1996;178(19):5803-5. Epub 1996/10/01. doi: 10.1128/jb.178.19.5803-5805.1996. PubMed PMID: 8824631; PubMed Central PMCID: PMCPMC178425.

33. Fralick JA, Burns-Keliher LL. Additive effect of *tolC* and *rfa* mutations on the hydrophobic barrier of the outer membrane of *Escherichia coli* K-12. J Bacteriol. 1994;176(20):6404-6. Epub 1994/10/01. doi: 10.1128/jb.176.20.6404-6406.1994. PubMed PMID: 7929014; PubMed Central PMCID: PMCPMC196984.

34. Piya D, Lessor L, Koehler B, Stonecipher A, Cahill J, Gill JJ. Genome-wide screens reveal *Escherichia coli* genes required for growth of T1-like phage LL5 and V5-like phage LL12. Sci Rep. 2020;10(1):8058. Epub 2020/05/18. doi: 10.1038/s41598-020-64981-7. PubMed PMID: 32415154; PubMed Central PMCID: PMCPMC7229145.

35. German GJ, Misra R. The TolC protein of *Escherichia coli* serves as a cell-surface receptor for the newly characterized TLS bacteriophage. J Mol Biol. 2001;308(4):579-85. Epub 2001/05/15. doi: 10.1006/jmbi.2001.4578. PubMed PMID: 11350161.

36. Rine J. A future of the model organism model. Mol Biol Cell. 2014;25(5):549-53. Epub 2014/03/01. doi: 10.1091/mbc.E12-10-0768. PubMed PMID: 24577733; PubMed Central PMCID: PMCPMC3937082.

37. Breitbart M, Bonnain C, Malki K, Sawaya NA. Phage puppet masters of the marine microbial realm. Nat Microbiol. 2018;3(7):754-66. Epub 2018/06/06. doi: 10.1038/s41564-018-0166-y. PubMed PMID: 29867096.
