# Supplementary figures and images for: Systematic exploration of Escherichia coli phage–host interactions with the BASEL phage collection
Source: PLoS Biol. 2021 Nov 16;19(11):e3001424. doi: 10.1371/journal.pbio.3001424 (PMC8594841; doi:10.1371/journal.pbio.3001424)

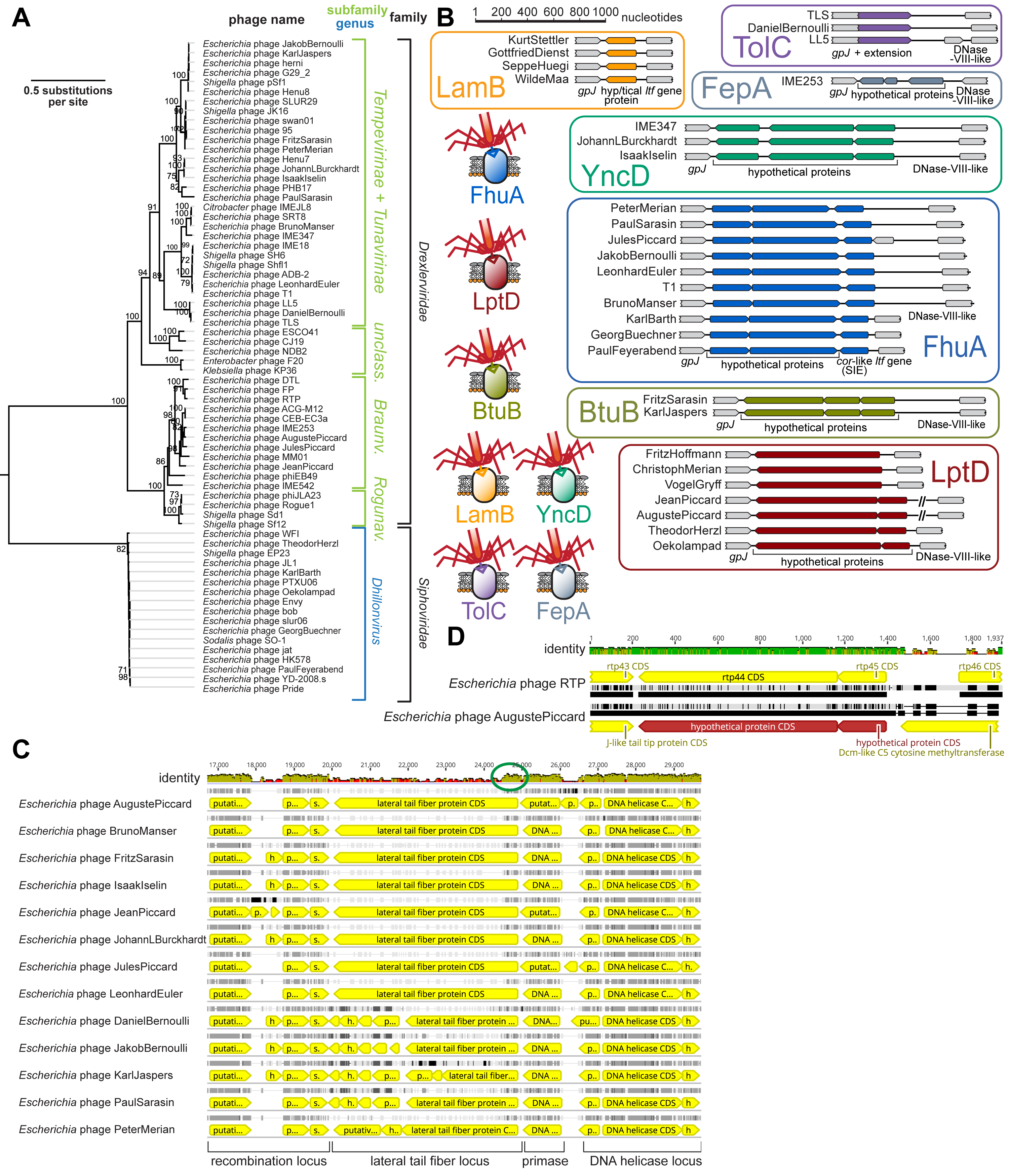

Supplement: S1 Fig — (A) Maximum-Likelihood phylogeny of Drexlerviridae and the Dhillonvirus genus of Siphoviridae based on several core genes with bootstrap support of branches shown if >70/100. It is clearly apparent that Drexlerviridae are split into 2 major clades, one formed by Braunvirinae and Rogunavirinae and another one formed by Tempevirinae, Tunavirinae, plus a few other groups. Given that the phylogenies strongly agree on all major branches, the root of the Drexlerviridae phylogeny shown in Fig 3D was placed between these 2 major clades. (B) The bona fide RBP loci downstream of the gpJ homolog are shown for all small siphoviruses (Drexlerviridae and Siphoviridae of Dhillonvirus, Nonagvirus, and Seuratvirus genera) where we had experimentally determined the terminal receptor (together with selected representatives where previous work had determined the receptor specificity). (C) The locus encoding lateral tail fibers was analyzed in a sequence alignment of the 13 Drexlerviridae phage genomes of the BASEL collection (see Materials and methods). It is clearly visible that the upstream and downstream regions (encoding genes involved in recombination as well as primase/helicase proteins for genome replication) are highly conserved and fully syntenic, with exception of small insertions in a few sequences. Conversely, only the most 5′ end of the largest lateral tail fiber protein gene is very similar among all analyzed genomes (green circle), while the rest shows neither synteny nor clear homology across all genomes. (D) The bona fide RBP locus of E. coli phage RTP was aligned to the homologous locus of phage AugustePiccard (Bas01) as described in Materials and methods. For the region comprising rtp44 and rtp45 of phage RTP, the pairwise identity of the 2 nucleotide sequences is ca. 93%. BASEL, BActeriophage SElection for your Laboratory; RBP, receptor-binding protein. (TIF) [file pbio.3001424.s011.tif]

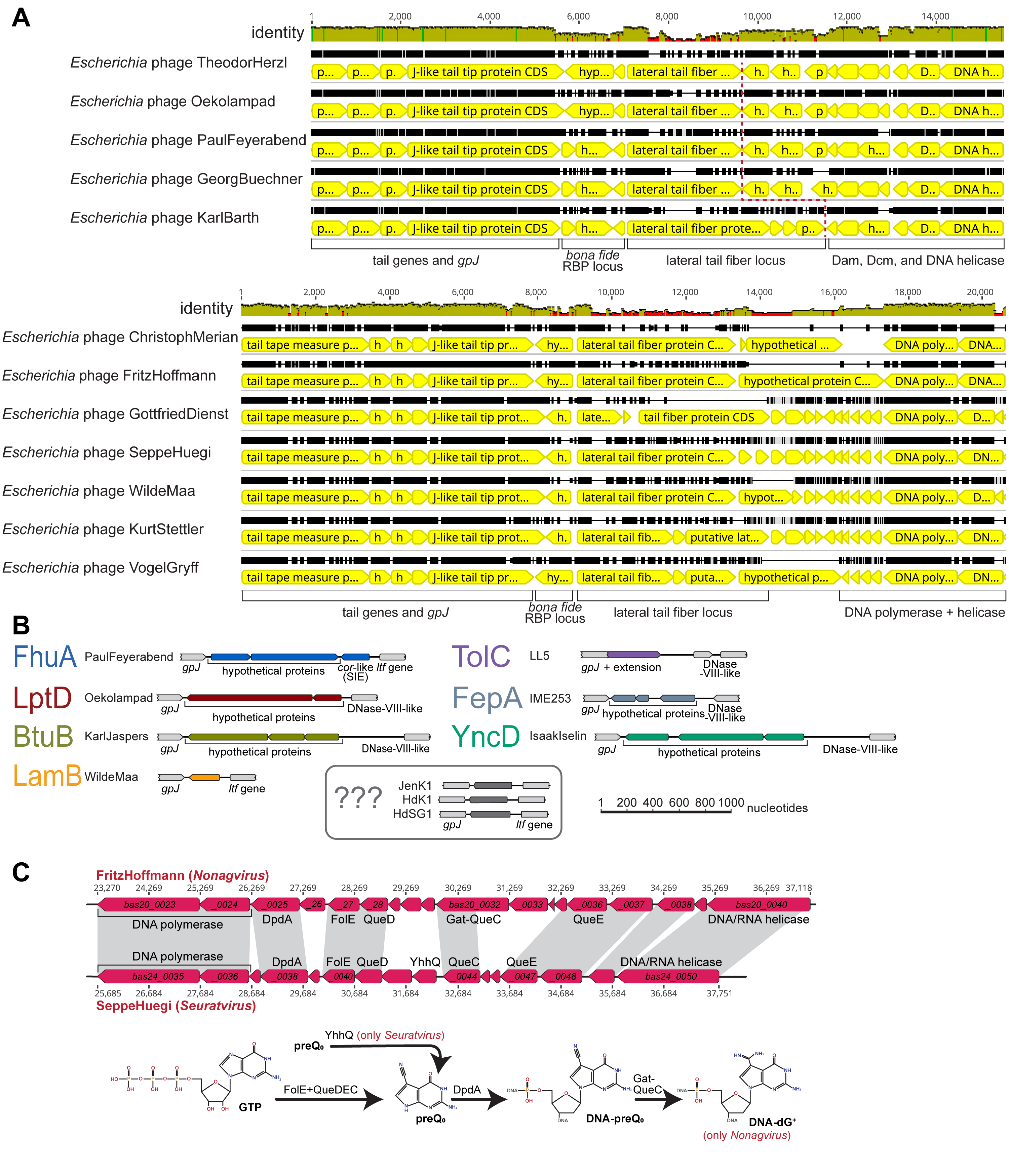

Supplement: S2 Fig — (A) The locus encoding lateral tail fibers was analyzed in sequence alignments of the 5 Dhillonvirus phage genomes of the BASEL collection (top) and the 7 Queuovirinae phage genomes (genera Nonagvirus + Seuratvirus; bottom) of the BASEL collection as described in Materials and methods. In both cases, 2 clear dips in overall sequence similarity are obvious, once at the bona fide RBP locus and then at the lateral tail fiber locus downstream of the far 5′ end of its first gene. (B) Schematic comparison of representative bona fide RBP loci as shown in S1B Fig to the corresponding allele of E. coli phages JenK1, HdK1, and HdsG1 that does not clearly match any of them. (C) Nonagvirus and Seuratvirus phages share a core 7-deazaguanosine biosynthesis pathway involving FolE, QueD, QueE, and QueC, which synthesizes dPreQ0 that is inserted into their genomes by DpdA. In Nonagvirus phages, the fusion of QueC with a Gat domain to Gat-QueC results in the modification with dG+ instead of dPreQ0 [43]. BASEL, BActeriophage SElection for your Laboratory; Gat, glutamate amidotransferase; RBP, receptor-binding protein. (TIF) [file pbio.3001424.s012.tif]

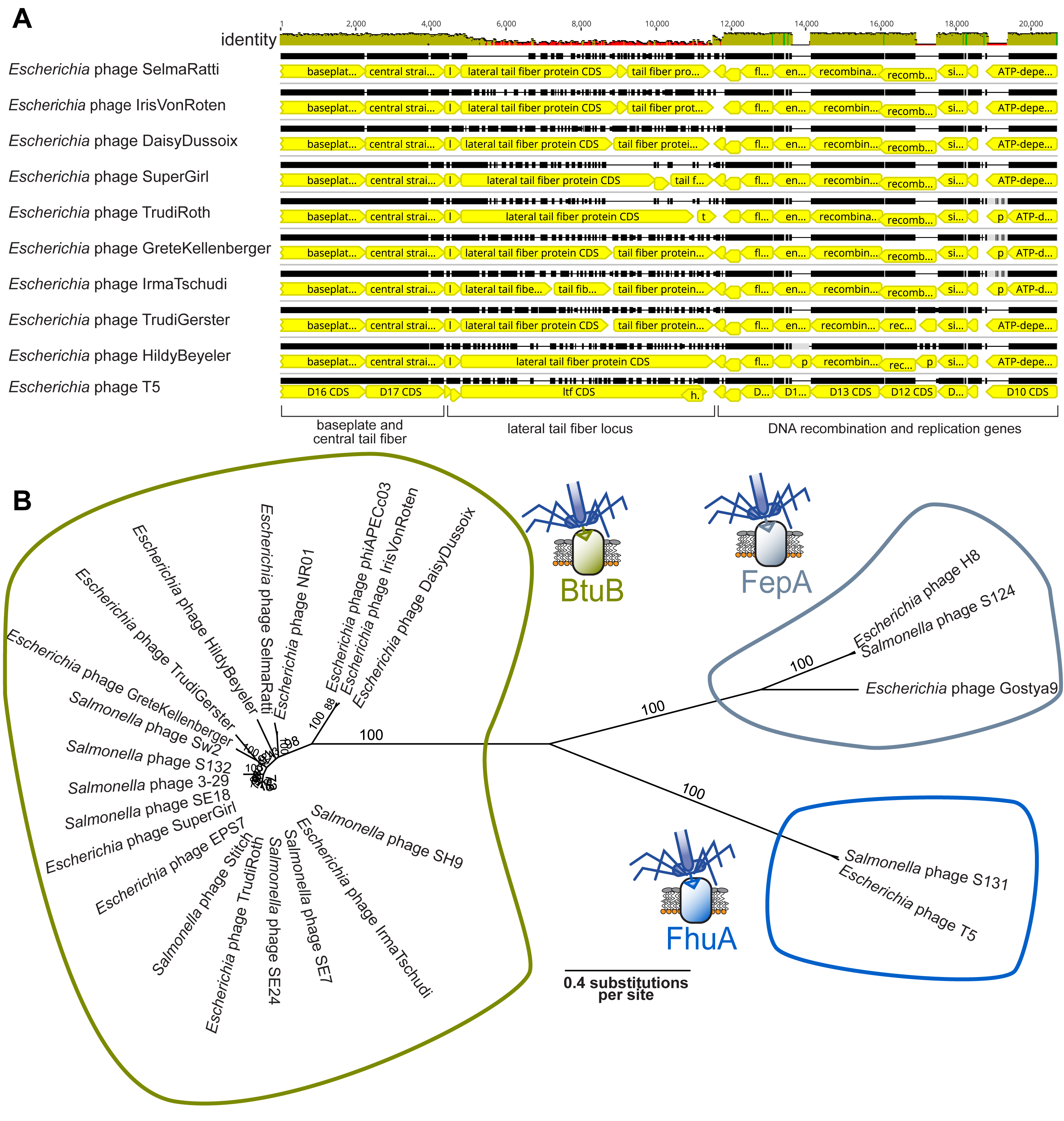

Supplement: S3 Fig — (A) The locus encoding lateral tail fibers was analyzed in a sequence alignment of the Demerecviridae: Markadamsvirinae phage genomes of the BASEL collection as described in Materials and methods. Sequence identity is high upstream and downstream of the lateral tail fiber locus (with exception of presence/absence of a few putative homing endonucleases) but drops considerably at the lateral tail fiber genes. Note that, as described previously, the lateral tail fibers can either be composed of a single large polypeptide or by 2 (or more) separate proteins [49,59]. The same diversity in architecture of lateral tail fibers can also be seen at the corresponding loci of small siphoviruses (S1C and S2A Figs). (B) The illustration shows a phylogeny of the RBPs of all Markadamsvirinae phages shown in Fig 6C. Briefly, the RBP genes of all genomes (invariably encoded directly upstream of the terminase genes) were translated, aligned, and then used to generate a phylogeny as described in Materials and methods. Three clear clusters emerge, one including all phages known to bind BtuB (left), one including all phages known to bind FepA (top right), and one including all phages known to bind FhuA (bottom right). Based on similar analyses by others [61], we conclude that the position of RBPs in these clusters is predictive of terminal receptor specificity of the phages encoding them. BASEL, BActeriophage SElection for your Laboratory; RBP, receptor-binding protein. (TIF) [file pbio.3001424.s013.tif]

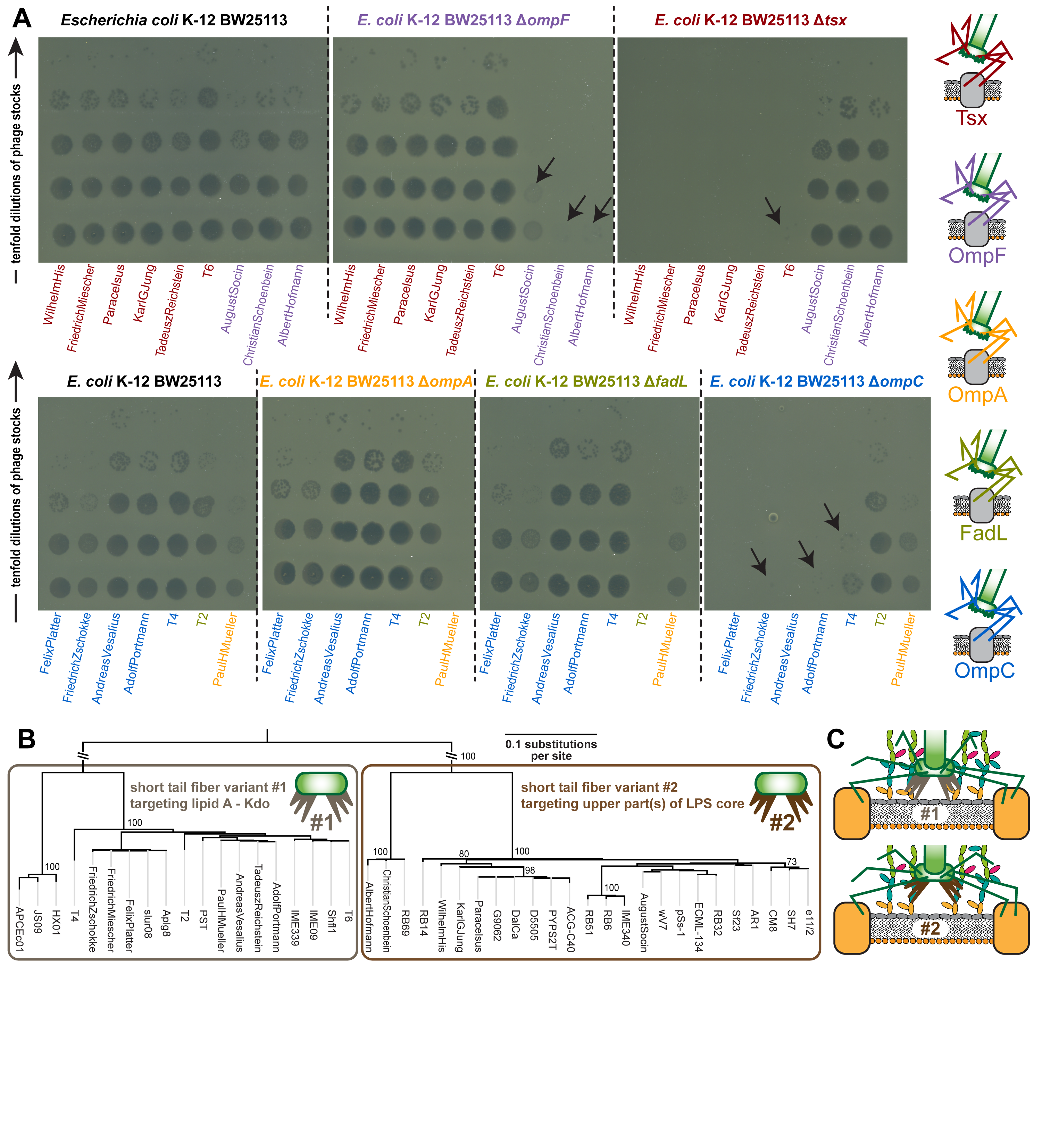

Supplement: S4 Fig — (A) Top agar assays with different surface protein mutants of the KEIO collection in comparison to the ancestral E. coli K-12 BW25113 strain were performed with serial 10-fold dilutions of all Tevenvirinae phages used in this study (undiluted phage lysates at the bottom and increasingly diluted samples toward the top). The phages show impaired growth on each one of the mutants, which identifies the primary receptor of each phage (also indicated by the color code highlighted on the right). Note that growth inhibition on the primary receptor mutants is rarely total and often still enables strongly reduced, heterogeneous plaque formation (arrows), especially after prolonged incubation of the top agar plates. (B) The Maximum-Likelihood phylogeny of Tevenvirinae short tail fiber proteins reveals 2 homologous, yet clearly distinct, clusters that correlate with the absence (variant #1, like T4) or presence (variant #2) of detectable LPS core dependence as shown in Fig 7E. (C) The results of (B) indicate that variant #1, as shown for T4, binds the deep lipid A–Kdo region of the enterobacterial LPS core, while variant #2 binds a more distal part of the (probably inner) core. LPS, lipopolysaccharide. (TIF) [file pbio.3001424.s014.tif]

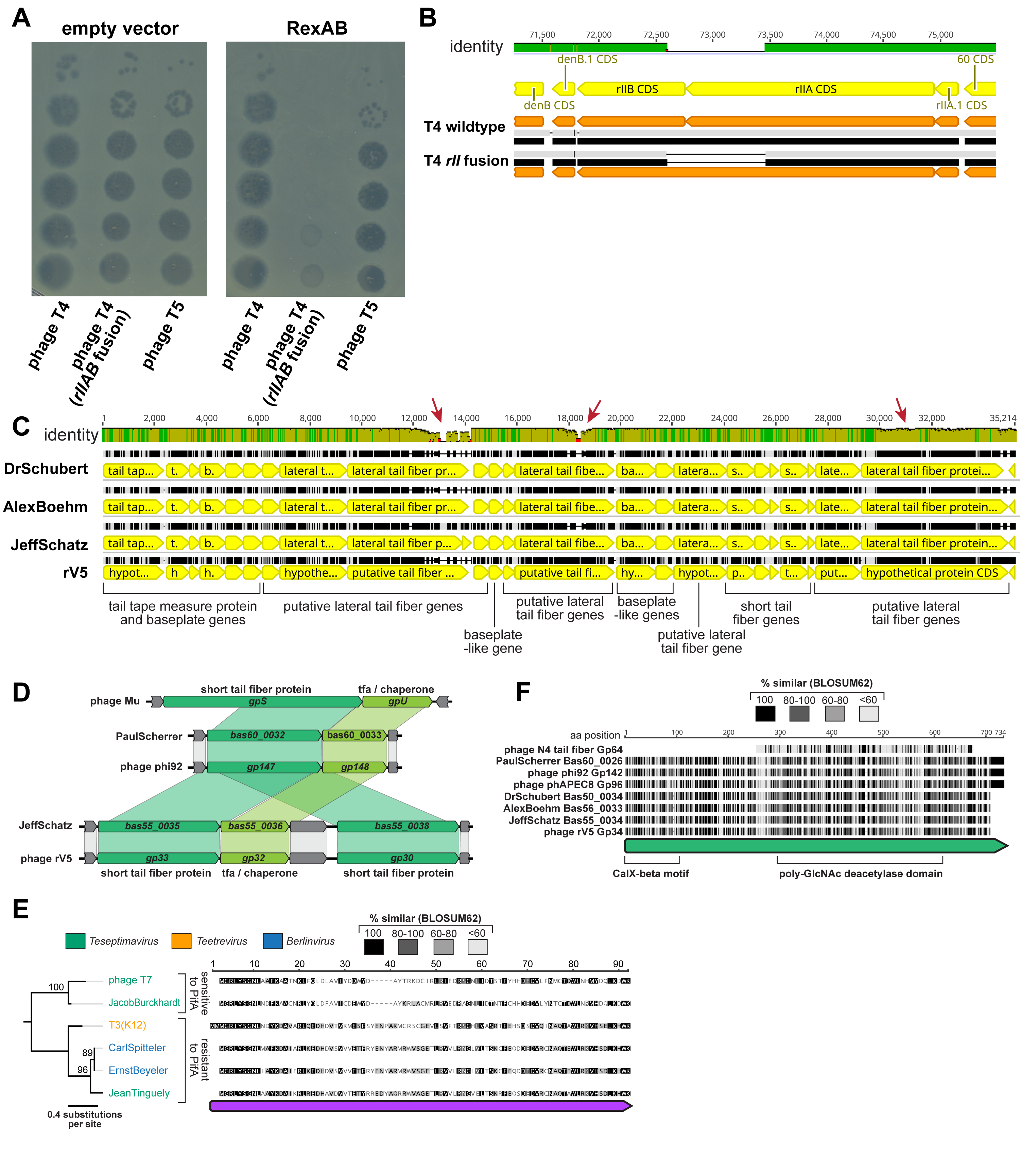

Supplement: S5 Fig — (A) Top agar assays of reference strain E. coli K-12 ΔRM carrying empty vector pBR322_ΔPtet or pAH213_rexAB were performed with serial 10-fold dilutions of phage T4 wild type, a T4 variant encoding an apparently hypomorphic rIIAB fusion (see (B)), and phage T5 (as control). The rIIAB mutant of phage T4 is unable to form plaques on the rexAB-expressing host and shows only “lysis from without” [97], while the T4 wild type and phage T5 are not affected. (B) A T4 phage mutant was erroneously obtained from a culture collection instead of the wild type and encoded a peculiar rII allele with fusion of the rIIA and rIIB open reading frames (shown as orange arrows; genome sequence determined by whole-genome sequencing as described in Materials and methods). Since such a mutant seems unlikely to arise spontaneously during shipping, we find it likely that this phage strain is related to the rIIAB fusion mutants employed for discovery of the triplet nature of the genetic code that were once commonly used (concisely reviewed in [152,153]). Notably, position and size of the deletion fusing rIIA and rIIB are indistinguishable from the sketch drawn by Benzer and Champe for the rIIAB fusion mutant r1589, which was used in the aforementioned work [154]. Unlike T4 wild type, the rIIAB fusion mutant is susceptible to rexAB when ectopically expressed from a plasmid vector (see (A)) and therefore validates functionality of the rexAB construct. (C) The illustration shows a sequence alignment of the locus encoding lateral tail fiber genes in phage rV5 and new isolates DrSchubert, AlexBoehm, and JeffSchatz that broadly cover the phylogenetic range of this genus (Fig 8C). It extends from the tail tape measure protein of phage rV5 (gp49) to the last large lateral tail fiber gene (gp27) [69,70]. Note that most genes are highly conserved including the lateral tail fiber component with sugar deacetylase domain (see (F); around position 8,000 in this alignment) or the short tail fiber locus (comp [file pbio.3001424.s015.tif]

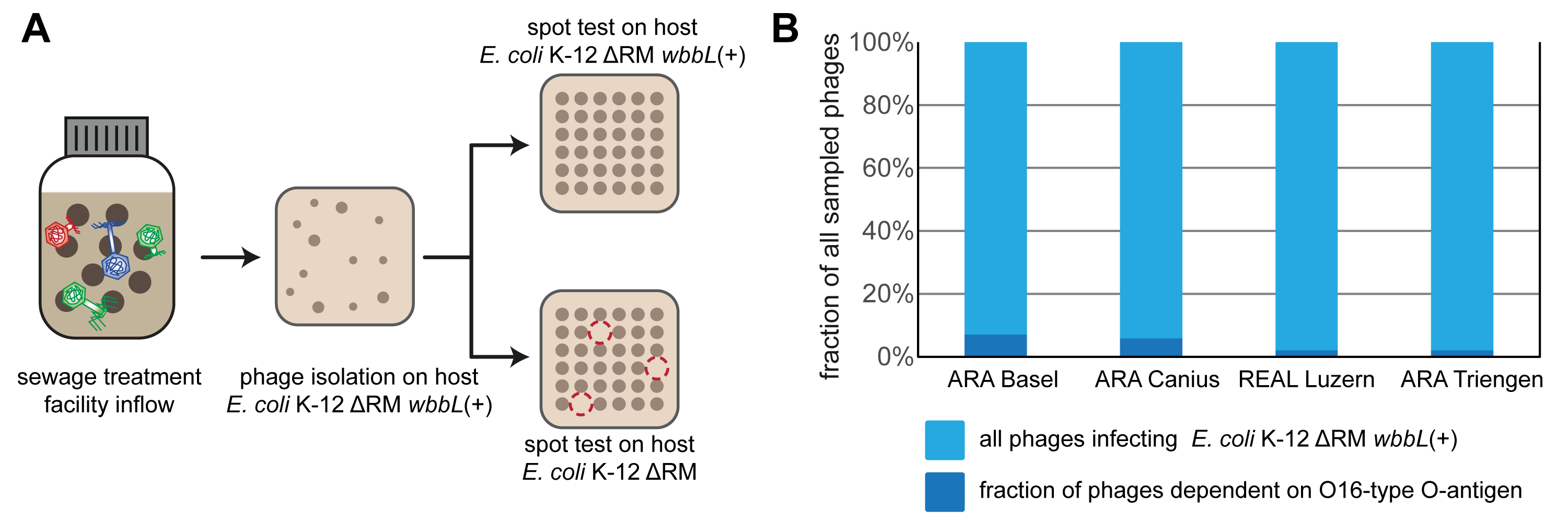

Supplement: S6 Fig — (A) Scheme showing how the fraction of bacteriophages depending on the O16-type O-antigen for each sewage treatment facility was quantified. Briefly, per facility, we first sampled 144 new phage isolates on E. coli K-12 MG1655 ΔRM wbbL(+) that expresses the O16-type O-antigen (Fig 2A). Subsequently, we performed qualitative top agar spot assays of these isolates on the same strain (growth control) and the parental E. coli K-12 MG1655 ΔRM that lacks O16-type O-antigen expression (Fig 2A). O-antigen–dependent isolates were be identified as those phages able to grow on the wbbL(+) host but not at all on the parental strain without O-antigen. (B) The fraction of bacteriophages that require the O16-type O-antigen for infectivity was very low for all sewage treatment facilities. Precise counts were 7.6% for ARA Basel (11/144), 6.3% for ARA Canius (9/144), and 2.1% for each REAL Luzern and ARA Triengen (3/144 in both cases). (TIF) [file pbio.3001424.s016.tif]

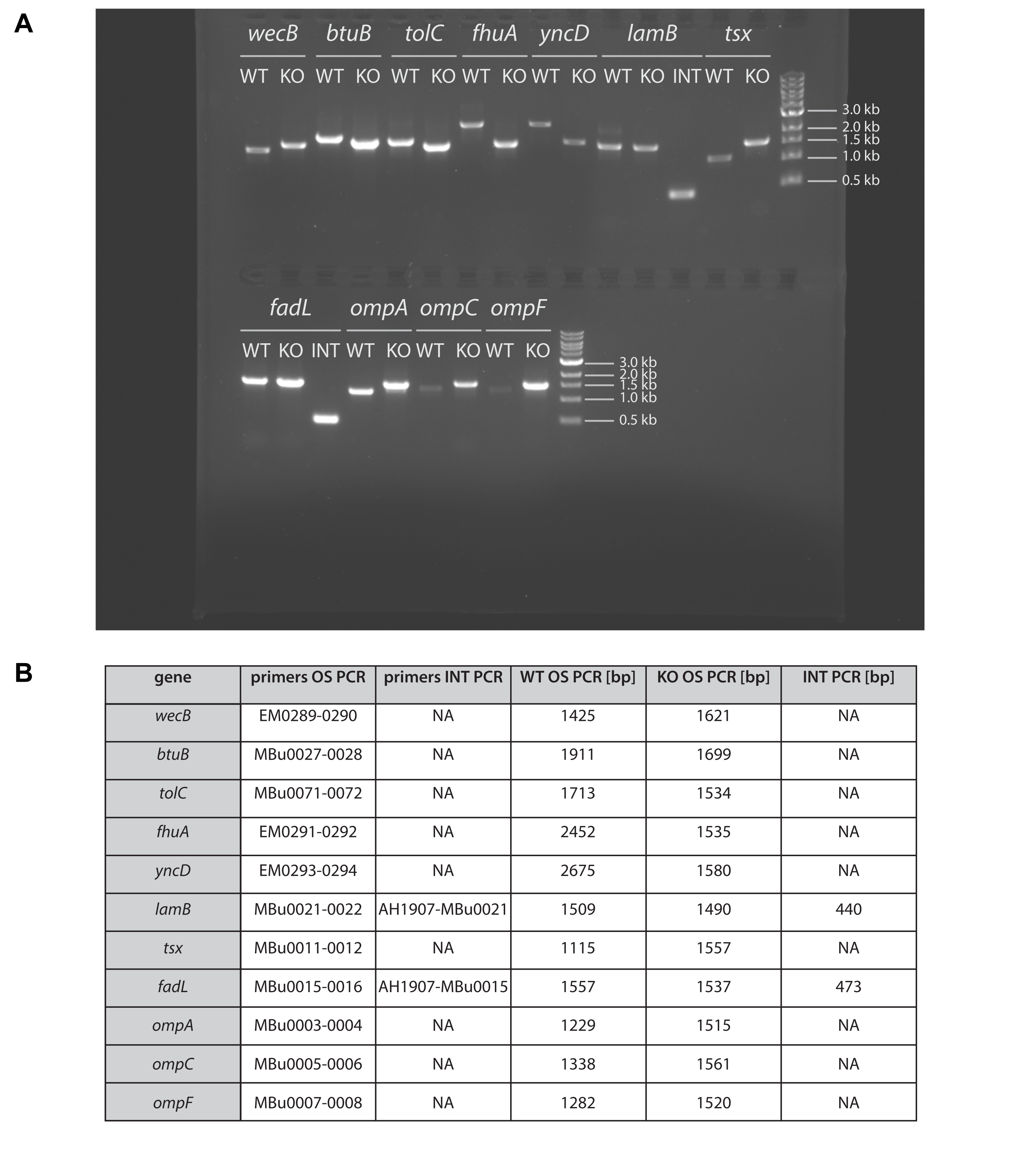

Supplement: S7 Fig — (A, B) The identity of all KEIO collection strains and isogenic mutants (btuB and tolC KOs; see Materials and methods and S1 Text) was probed by diagnostic PCR over the gene that should have been replaced with a kanamycin resistance cassette [118] and subsequent agarose gel electrophoresis of the PCR products. Panel (A) shows the agarose gel with all PCR products and, for reference, the “1 kb DNA ladder” of New England Biolabs (right). Panel (B) lists the oligonucleotide primers used for all PCRs (sequences compiled in S2 Table) and the expected sizes of PCR products for the OS PCRs of WT and KO alleles. In cases where WT and KO alleles were expected to have a similar size, we performed an additional PCR with 1 INT primer that anneals inside the kanamycin resistance cassette and 1 primer at the target locus. These PCRs can only result in a PCR product if the correct mutant strain was used as PCR template. The agarose gel in (A) confirms the identity of all KEIO collection KOs and isogenic strains that we used in our study. INT, internal; KO, knockout; OS, overspanning; WT, wild-type. (TIF) [file pbio.3001424.s017.tif]

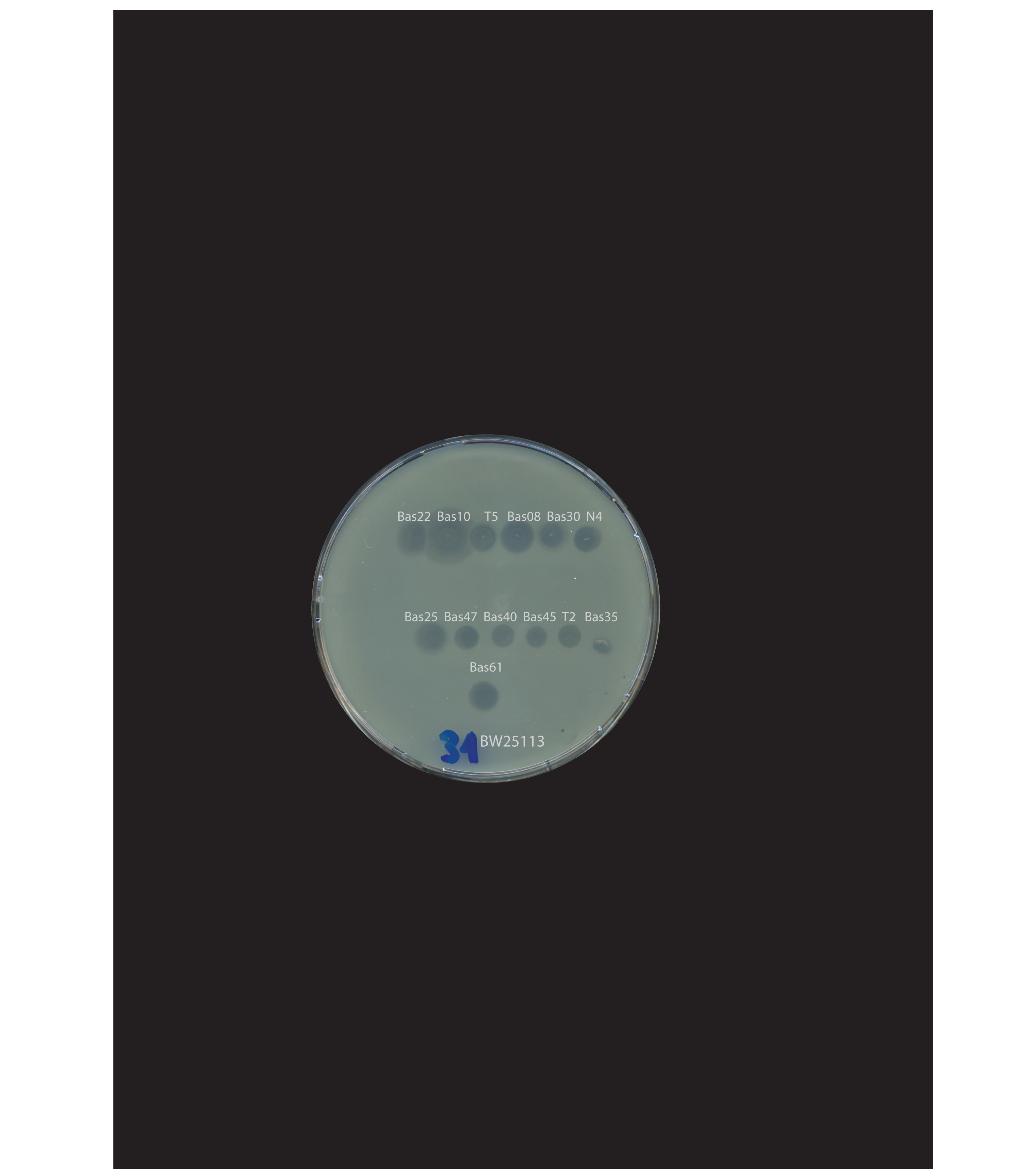

Supplement: S8 Fig — Thirteen phages were chosen to confirm the phenotypes of all E. coli mutants used for bacteriophage receptor identification by genetic complementation in qualitative top agar spot assays (S9–S13 Figs). These were N4 (bona fide ECA-targeting phage dependent on wecB), TrudiRoth (Bas30; targets BtuB), DanielBernoulli (Bas08; targets TolC), T5 (targets FhuA), IsaakIselin (Bas10; targets YncD), KurtStettler (Bas22; targets LamB), WilhelmHis (Bas35; targets Tsx), T2 (targets FadL), PaulHMueller (Bas45; targets OmpA), FelixPlatter (Bas40; targets OmpC), AlbertHofmann (Bas47; targets OmpF), VogelGryff (Bas25; targets LptD), and EmilieFrey (Bas61; bona fide ECA-targeting phage dependent on wecB). Several phages additionally depend on the LPS core and therefore on waaC and sometimes also waaG for their infectivity (see Fig 2A). These were DanielBernoulli (Bas08), WilhelmHis (Bas35), AlbertHofmann (Bas47), VogelGryff (Bas25), and EmilieFrey (Bas61). The top agar plate shown in this figure was generated with the parental E. coli K-12 BW25113 wild-type control that was readily infected by all phages. LPS, lipopolysaccharide. (TIF) [file pbio.3001424.s018.tif]

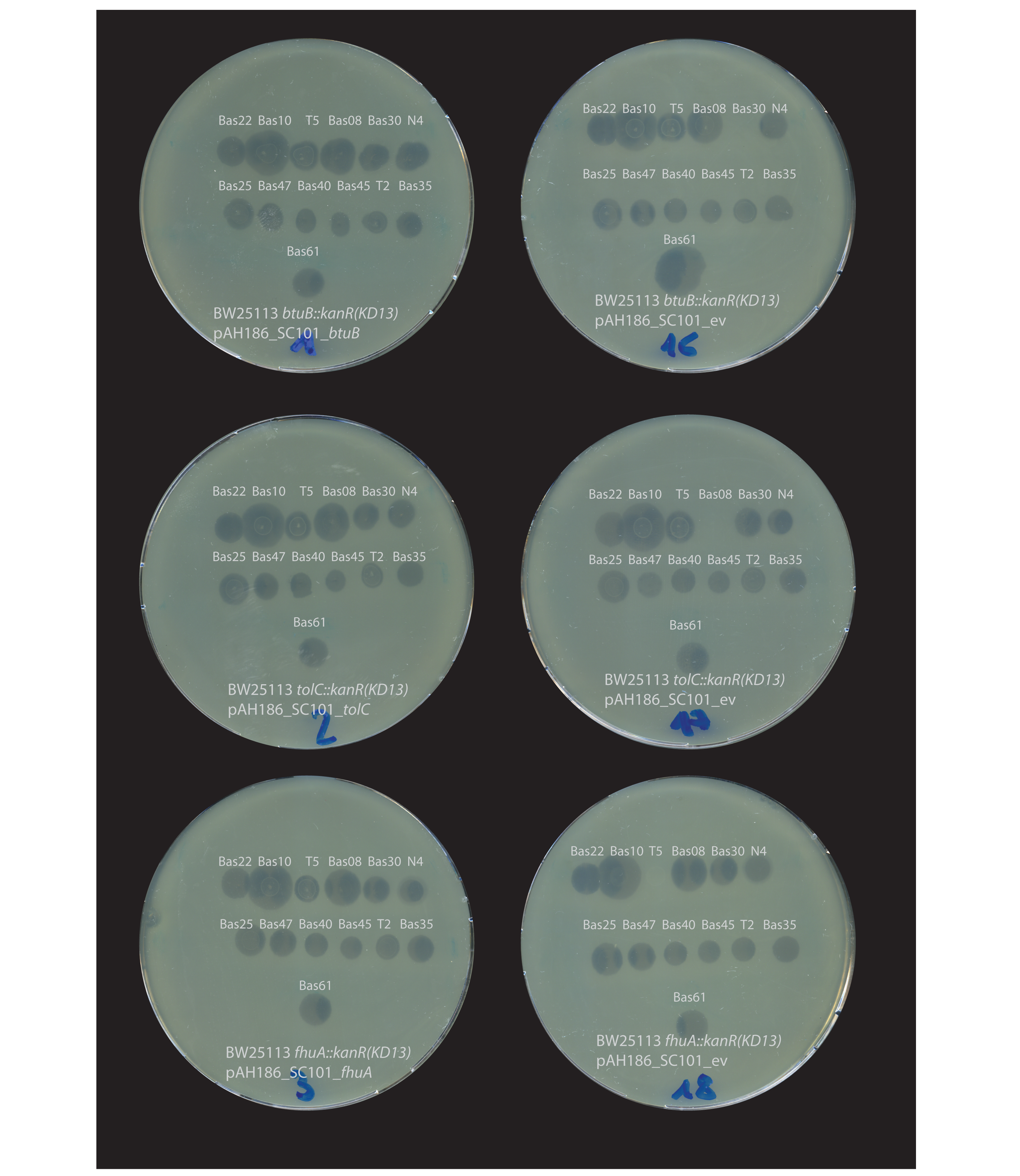

Supplement: S9 Fig — Top agar spot assays with the 13 phages highlighted in S8 Fig were performed with the btuB (top), tolC (middle), and fhuA (bottom) mutant strains of E. coli K-12 BW25113. The plates on the left side were generated with strains carrying the respective complementation plasmid (see Materials and methods) and the plates on the right side with E. coli carrying the ev control. The plates show that the btuB dependency of TrudiRoth (Bas30; top), the tolC dependency of DanielBernoulli (Bas08; middle), and the fhuA dependency of T5 (bottom) could be fully complemented. ev, empty vector. (TIF) [file pbio.3001424.s019.tif]

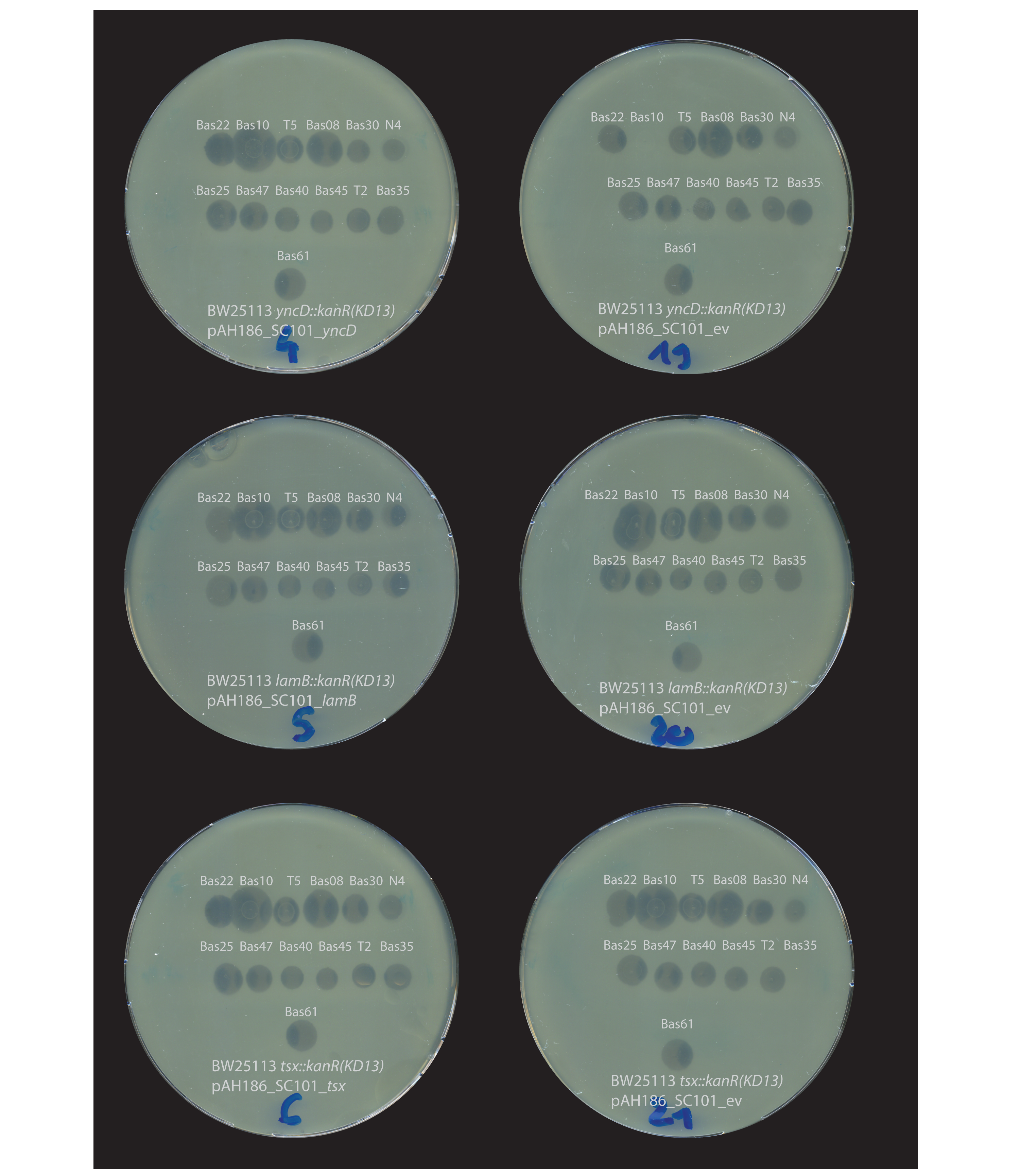

Supplement: S10 Fig — Top agar spot assays with the 13 phages highlighted in S8 Fig were performed with the yncD (top), lamB (middle), and tsx (bottom) mutant strains of E. coli K-12 BW25113. The plates on the left side were generated with strains carrying the respective complementation plasmid (see Materials and methods) and the plates on the right side with E. coli carrying the ev control. The plates show that the yncD dependency of IsaakIselin (Bas10; top), the lamB dependency of KurtStettler (Bas33; middle), and the tsx dependency of WilhelmHis (Bas35; bottom) could be fully complemented. ev, empty vector. (TIF) [file pbio.3001424.s020.tif]

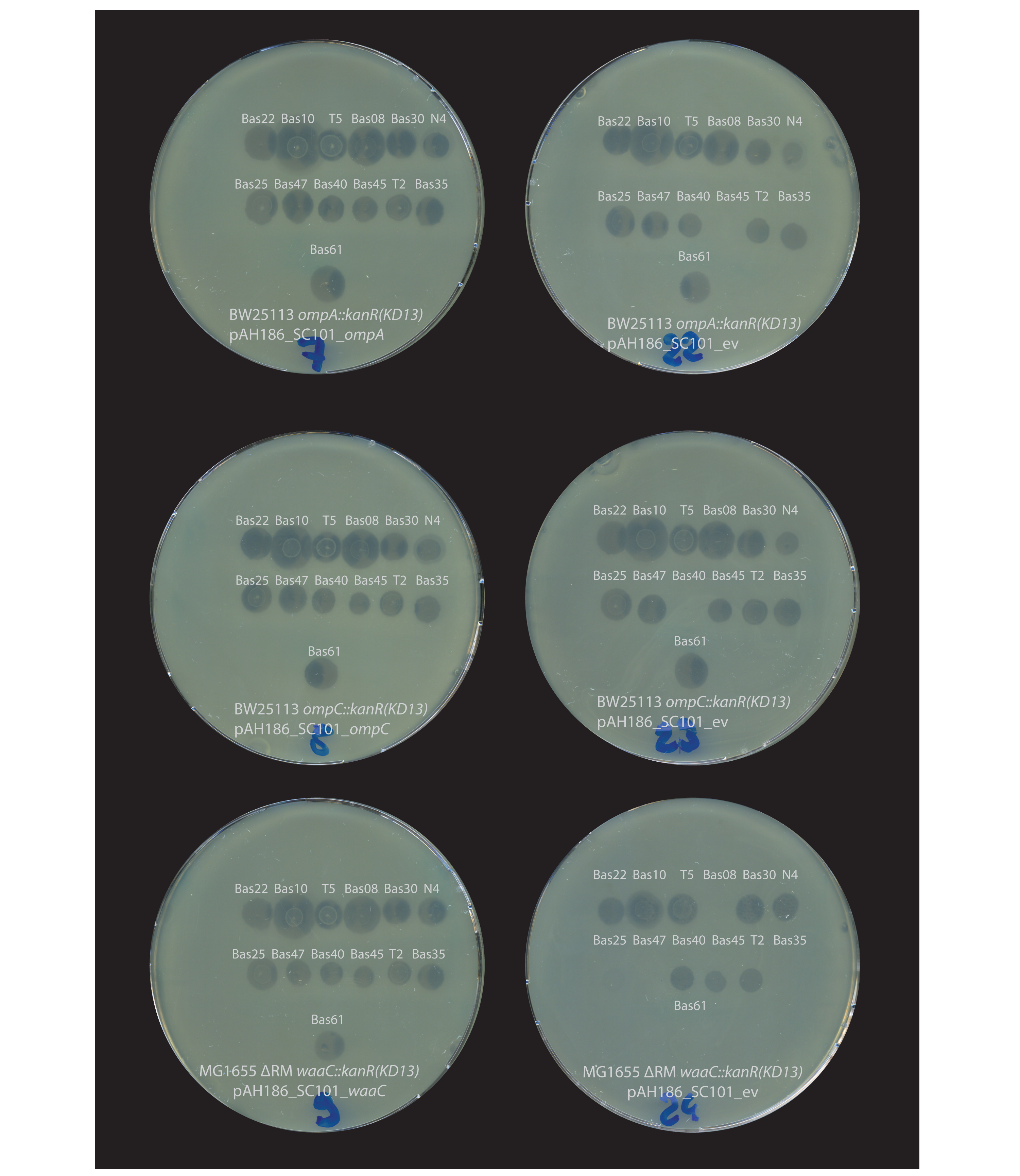

Supplement: S11 Fig — Top agar spot assays with the 13 phages highlighted in S8 Fig were performed with the ompA (top) and ompC (middle) mutant strains of E. coli K-12 BW25113 as well as the waaC mutant of E. coli K 12 MG1655 ΔRM (bottom). The plates on the left side were generated with strains carrying the respective complementation plasmid (see Materials and methods) and the plates on the right side with E. coli carrying the ev control. The plates show that the ompA dependency of PaulHMueller (Bas45; top) and the ompC dependency of FelixPlatter (Bas40; middle) could be fully complemented. Similarly, the loss of infectivity of DanielBernoulli (Bas08), WilhelmHis (Bas35), AlbertHofmann (Bas47), VogelGryff (Bas25), and EmilieFrey (Bas61) on the waaC mutant could be fully complemented by genetic complementation (bottom). ev, empty vector. (TIF) [file pbio.3001424.s021.tif]

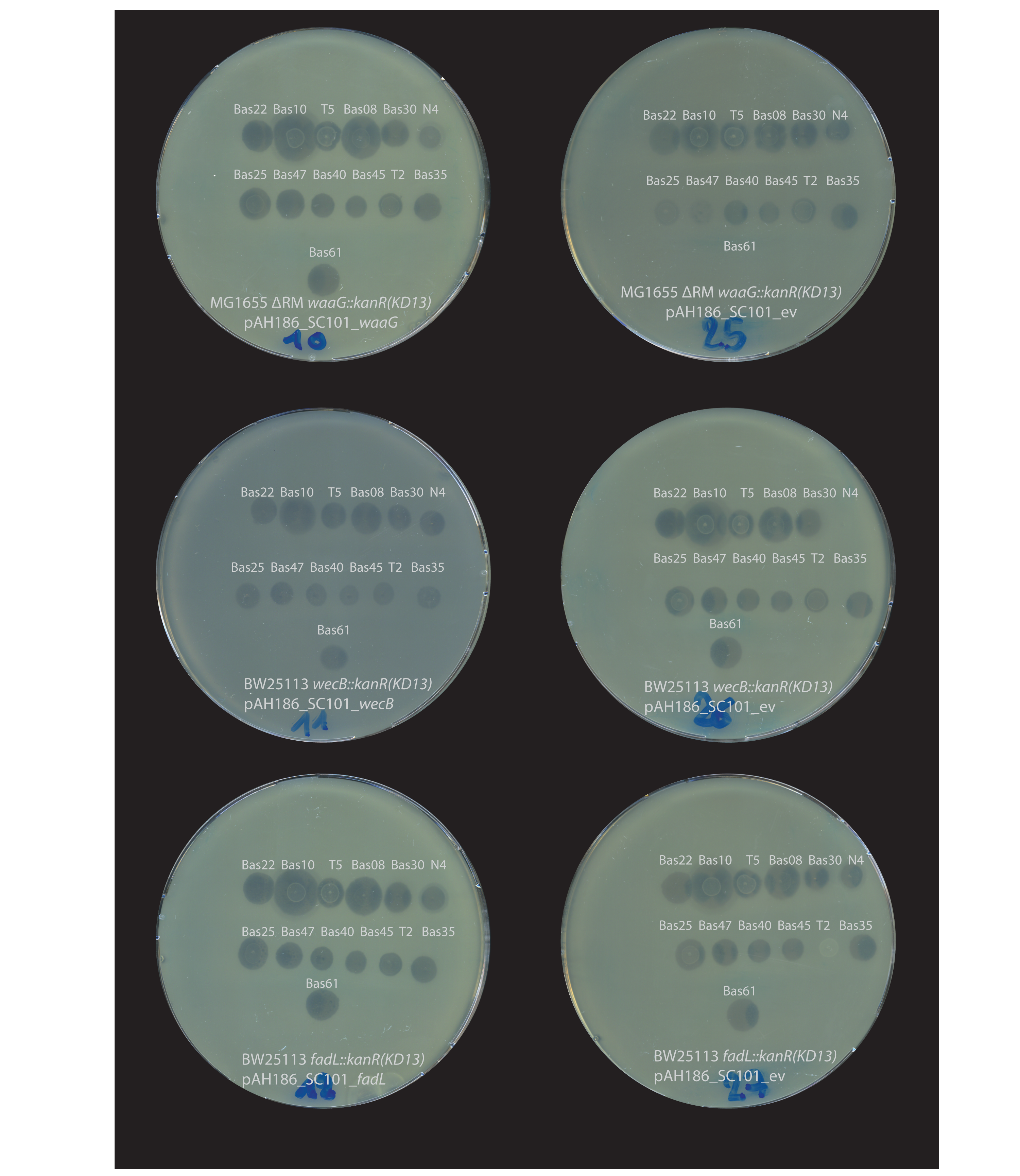

Supplement: S12 Fig — Top agar spot assays with the 13 phages highlighted in S8 Fig were performed with the waaG mutant of E. coli K 12 MG1655 ΔRM (top) and the wecB (middle) as well as fadL (bottom) mutants of E. coli K-12 BW25113. The plates on the left side were generated with strains carrying the respective complementation plasmid (see Materials and methods) and the plates on the right side with E. coli carrying the ev control. The plates show that the total loss of infectivity of EmilieFrey (Bas61) as well as the clearly reduced infectivity of AlbertHofmann (Bas47) and VogelGryff (Bas25) on the waaG mutant could be fully complemented by genetic complementation (top). Similarly, the wecB dependency of N4 (middle) and the fadL dependency of T2 (bottom) could be fully complemented. Unlike for all other complementation constructs, full complementation with the wecB plasmid required induction of expression driven by the Plac on pAH186SC101 with 1 mM of IPTG during the overnight culture. ev, empty vector; IPTG, isopropyl β-d-1-thiogalactopyranoside. (TIF) [file pbio.3001424.s022.tif]

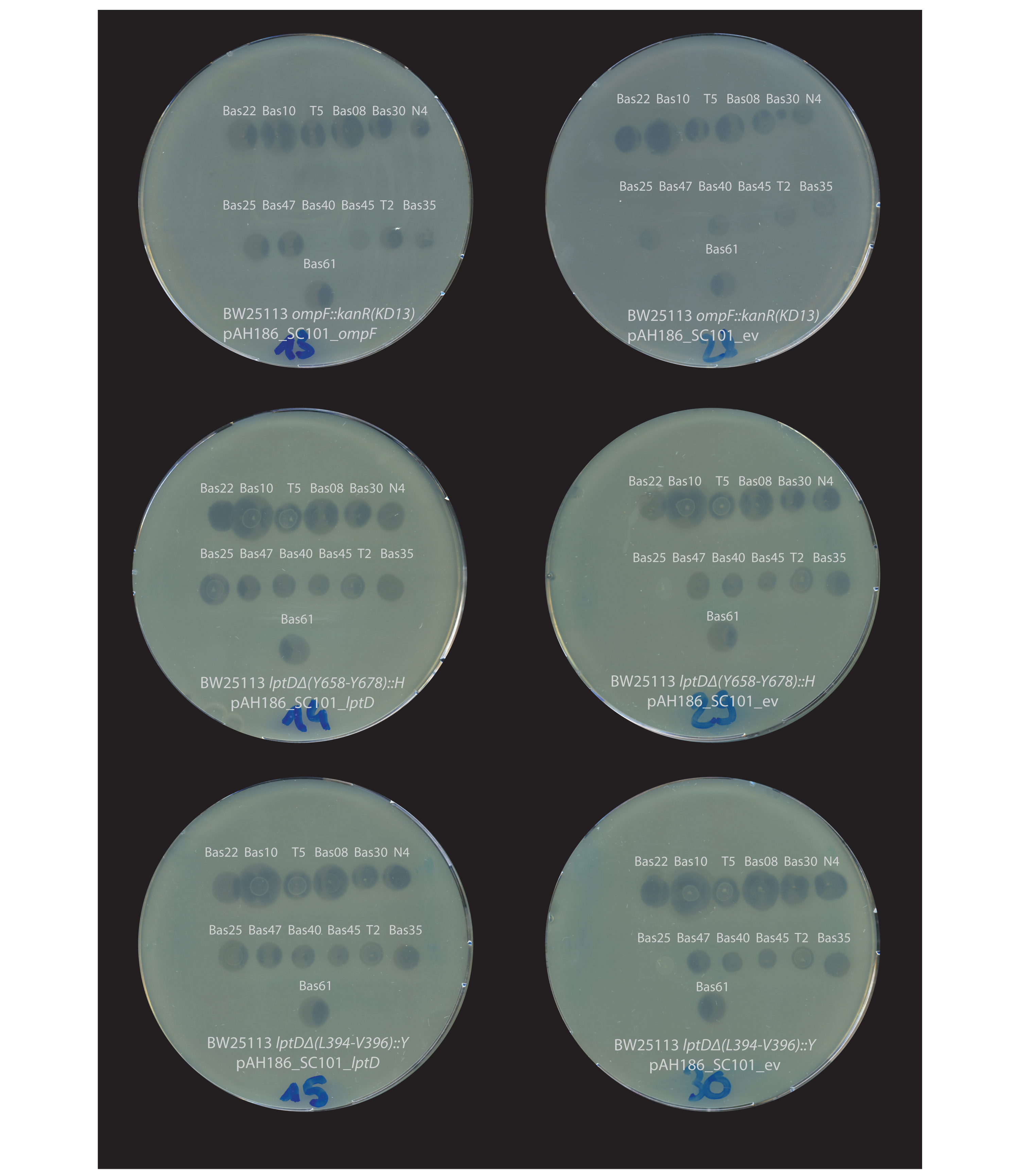

Supplement: S13 Fig — Top agar spot assays with the 13 phages highlighted in S8 Fig were performed with the ompF knockout (top) as well as the lptDΔ(Y658-Y678)::H (middle) and lptDΔ(L394-V396)::Y mutants (middle and bottom plates; see also Fig 4C and 4D). The plates on the left side were generated with strains carrying the respective complementation plasmid (see Materials and methods) and the plates on the right side with E. coli carrying the ev control. The plates show that the ompF dependency of AlbertHofmann (Bas47; top) and the lptD dependency of VogelGryff (Bas25; middle and bottom plates) could be fully complemented. Curiously, complementation of the ompF mutant abolished infections by FelixPlatter (Bas40; compare left and right plates of the top panel). A similar phenomenon was previously observed by others who reported that ompF overexpression inhibited infections by bacteriophage T4, possibly by indirectly interfering with expression of its primary receptor OmpC that is also the primary receptor of FelixPlatter (Bas40; see Figs 7C and 7D and S4A) ([72] and literature cited therein). ev, empty vector. (TIF) [file pbio.3001424.s023.tif]
