# Supplementary material for: Systematic exploration of Escherichia coli phage–host interactions with the BASEL phage collection
Source: PLoS Biol. 2021 Nov 16;19(11):e3001424. doi: 10.1371/journal.pbio.3001424 (PMC8594841; doi:10.1371/journal.pbio.3001424)
Supplement: Attachment — Submitted filename: reviewerresponse.docx [file pbio.3001424.s024.docx]

**Point-by-point response to referees/editor’s comments**

**PBIOLOGY-D-21-00722R1**

We are grateful for the overall constructive and helpful comments of reviewers and editor that we have now addressed in a revised manuscript. To improve accessibility and readability of the manuscript, we also followed the advice of reviewers and editor to shorten the main text by ca. 10% (from 10'926 words introduction-to-discussion down to 9’978 words). While preparing the revised manuscript, we also added two new representatives of the phi92-like group of phages (relatives of *Vequintavirinae*) that have by far the largest lysis host range of all tested phages which we anticipate to be a target of future work on the BASEL collection. Together with phage PaulScherrer (Bas60) that had already been part of the original submission, this extends the coverage of this group to three isolates (see the phylogeny of Fig 8C of the revised manuscript).

In the following, we reply to the reviewers’ comments and concerns one-by-one and outline in detail how we have addressed them:

**Referees' comments – Reviewer #1**

*As reviewers, we are supportive of the intent of the paper; however, we do not feel that there is a conceptual advance made that warrants publication in PLOS Biology. The goal of developing a diverse resource collection that can be interrogated to inform basic phage biology is commendable. Unfortunately, in its current presentation - the figures and text are far too dense to appreciate what has been done. There probably are important insights in this paper, but it is too difficult to find these insights. The paper is overwhelming and difficult to read. One major barrier to the readability is the nomenclature - the taxonomy is being used too frequently, given that these words are not intuitive, difficult to say, (and likely will change) I suggest that the authors do not put so much emphasis on the taxonomy.*

We thank reviewer #1 for this critical yet constructive assessment of our work. This reviewer is certainly right (as also pointed out by the other reviewers) that figures and text of the original submission were too dense. We have therefore streamlined the manuscript and decluttered the illustrations (see below). Conversely, we do not fully agree with reviewer #1 that “taxonomy is being used too frequently”, and this view does also not seem to be shared by the other reviewers either - see, e.g., how reviewer #2 explicitly highlights in the first comment that we use the current viral taxonomy. Generally, it’s true that viral taxonomy is a moving target, but we do not know any other accurate and precise way to name bacteriophages besides the nomenclature that is approved by the International Committee on Taxonomy of Viruses (ICTV). This taxonomy certainly has its shortcomings and gaps, but some of these are explicitly highlighted by our work to constructively suggest improvements (e.g., the lack of a formal name for the group of phi92-like relatives of *Vequintavirinae* that are the sister clade of the *Phapecoctavirus* genus). Nevertheless, reviewer #1 certainly has a point that abundant use of formal taxonomic categories might discourage non-expert readers from going through our paper. In the revised manuscript we have therefore lightened the use of formal taxonomy by increasing the use of established informal synonyms such as “T-even phages” for *Tevenvirinae*.

*The first few sections of the results: Composition of BASEL Collection and 2 'overview' sections, do not actually contain results and do not belong here; this information can be distilled down to be included in the intro.*

*Figures 1-3 - do not actually contain much data (with the exception of a small panel in fig 2B).*

Reviewer #1 highlights the absence of truly new results from the first parts of our *Results* section and suggests major structural changes by removing these parts and by “distill[ing] down” this information into the introduction. In the revised manuscript we have now removed one figure (Fig 1 of the original submission) and streamlined the sections of text that had been targeted by this reviewer. However, no major cuts have been made because we feel that this part is important for enabling non-expert readers among the broad readership of PLoS Biology to understand the methodology of our work.

*The bait strain here lacked O antigen - this was confusing - this means no phages dependent on O antigen could be isolated. However, they acknowledge that phages may depend on O antigen. Page 6, line 111 - the description of this mutant would suggest you would not get any O-antigen dependent phages, which they did (i.e., the T5-like phages), so I'm just left confused.*

This comment of reviewer #1 (and a similar comment of reviewer #3) targets the lack of O-antigen expression by our *E. coli* K-12 bait strain for phage isolation which is a true yet deliberate limitation of our phage sampling. As this reviewer acknowledges, we have already discussed this issue in our original submission (now extended, e.g., in S2 Text of the revised manuscript).

Part of this reviewer’s comment is due to a misunderstanding about the relationship between phages and their host’s O-antigen. Notably, all laboratory strains of *E. coli* (K-12 and B lineages) lack O-antigen expression (see Fig 12 of the original submission / Fig 11 of the revised manuscript) and, consequently, the whole body of fundamental *E. coli* phage research has so far been done on hosts without O-antigen. Rather than that we would have arbitrarily picked a mutant without O-antigen, our work is therefore actually an exception in that we explicitly acknowledge the absence of O-antigen on these strains and systematically test the ability of isolated phages to overcome the O-antigen barrier. To take up the example highlighted in the reviewer comment, none of the tested T5-like phages *depends* on the O-antigen in any way, but one of them (IrisVonRoten) is simply not blocked by restoration of O-antigen expression in *E. coli* K-12 (Fig 7C in the original submission / Fig 6E in the revised manuscript).

Nevertheless, reviewer #1 is right that our strategy of phage isolation with a bait lacking O‑antigen will miss all phages that depend on O-antigen glycans for host recognition. We have already discussed this issue at several places in our original submission and to considerable detail on the first two pages of S3 Text (which is now S2 Text in the revised manuscript). As already outlined there based on isolation experiments of others, we see no evidence for the implicit assumption of reviewers #1 and #3 that these O‑antigen-dependent phages could be quantitatively or qualitatively important for our study.

O-antigen-dependent phages infecting *E. coli* are poorly studied and, to the best of our knowledge, no published work has ever quantified the abundance of O-antigen-dependent phages infecting any strain of this organism. To directly address this important comment of reviewer #1, we now performed additional phage isolation experiments to determine how many of those phages *that are able to* infect our *E. coli* K-12 strain with restored O16-type O-antigen actually *depend* on O-antigen expression for infection. Across sewage plant inflow from four difference treatment facilities, the proportion of these O-antigen-dependent *E. coli* phages is consistently very low (few percent of all isolates; see S6 Fig of the revised manuscript). These results are now presented in the first section of the *Discussion* and more comprehensively discussed in S2 Text of the revised manuscript.

*The following comment applies in general to many figures but is using Fig 7 as an example: the data itself in this figure (i.e., Fig 7c) is too small, the phylogenetic tree could be supplemental, I'm not sure about panel A - why is this here? The data pertaining to the receptors (depicted in the far-right panel) is not even shown on this figure - why is a schematic cartoon more important than the actual data?*

*Callouts explaining background on figures is odd for a primary research article and were reminiscent of figures more appropriate for a review article. Perhaps the authors can consider publishing a more clear, succinct version of the manuscript focusing on the data collected and provide a commentary piece/review type article elsewhere.*

We thank reviewer #1 for this comment that highlights the lack of clarity inherent to some design features of the illustrations in our original submission. In our revised manuscript we have addressed this point by redesigning our illustrations with a focus on clear data presentation. For all figures showing genomic and phenotypic data of different groups of phages (Figs 3 + 5-10 in the revised manuscript / Figs 4 + 6-11 in the original submission) we therefore increased the size of the bar diagrams with our phenotypic data and of the phylogenies that are essential for defining the identity of each phage isolate in relation to other phages. In addition, we shifted all more specific results to the supplemental figures and (in response to a comment of reviewer #3) added representative transmission electron micrographs of virions to each figure. The illustrations of bacteriophage adsorption and DNA injection in panel A of all these figures were kept because we feel that they increase accessibility of the data to non-expert readers.

**Referees' comments – Reviewer #2**

*The manuscript by Maffei et al represents a large amount of work on a collection of phages infecting a model organism, E. coli K-12. The Phages are sequenced and classified, and presented as groups using the new ICTV taxonomy. The phages are comprehensively evaluated for their receptor binding protein content, sensitivity to deletions in surface feature genes and anti-phage systems, and host range. A number of common features are noted among phage groups and this work represents a comprehensive and high-impact study that will aid in rationalizing at least a subsection of the large volume of phage genome sequence available. I have several comments and suggestions below that would strengthen the overall work and its presentation.*

We thank reviewer #2 for this favorable summary and evaluation of our work.

*GENERAL COMMENTS*

*In the methods, were the Keio collection mutants confirmed for their genotypes (e.g., by PCR)? Keio mutants have a reputation for not always being as advertised.*

Reviewer #2 is certainly right that the identity of Keio collection mutants should always be verified, e.g., by diagnostic PCR over the locus that had been targeted in each mutant. The large-scale analysis of different problems with Keio mutants by Yamamoto et al. (*Mol Syst Biol*, 2009) found no issues with the original construction of the mutant strains that we used. However, this does of course not exclude any cases of, e.g., mistaken identity on the trajectory from the original construction to our experiments.

In our revised manuscript we therefore now include a supplemental figure (S7 Fig) showing the results of diagnostic PCRs for every Keio collection mutant used in our manuscript (always in direct comparison to the parental wildtype *E. coli* K-12 BW25113). In cases where the predicted sizes of PCR products for the wildtype allele and the mutant allele were very similar, we included an additional PCR with one primer annealing inside the Keio collection kanamycin resistance cassette and one primer annealing outside the deletion at the target locus. This PCR can only result in a PCR product band if the correct Keio collection mutant is the template. Taken together, the results shown in S7 Fig of the revised manuscript unambiguously confirm the identity of all mutants.

*Also in the methods, were any of the single-gene deletions complemented to confirm the phenotype. This is not described in the methods but is standard practice when studying the phenotypes associated with gene knockouts.*

We thank reviewer #2 for this suggestion to demonstrate that the phenotypes of all used *E. coli* mutants are truly due to each mutant’s nominal genotype and not, e.g., due to polar effects of the Keio collection kanamycin resistance cassette or suppressor mutations. As proposed by the reviewer, we have addressed this issue by complementing all *E. coli* mutants used in this study with plasmids encoding a wildtype copy of the respective deleted or otherwise altered gene of each mutant. These data are included in the revised manuscript as a set of supplemental figures (S8 - S13 Figs). Every single mutant could be functionally complemented.

*Use caution when interpreting results in mutants with deep LPS core defects. The "deep rough" phenotype has been associated with pleiotropic effects including derangement in LPS structure and proper placement of porins in the outer membrane, which could affect phage sensitivity in multiple ways. This is also true of TolC mutants, which appear to have improperly processed LPS and similar outer membrane defects.*

We thank reviewer #2 for highlighting pleiotropic cell envelope phenotypes of our *waaC* and *waaG* LPS core mutants as well as the *tolC* knockout. This comment was addressed comprehensively in a dedicated section “Considerations regarding the phenotyping of knockout mutants with pleiotropic outer membrane phenotypes” of the revised version of S2 Text. Briefly, while it is important to be cautious with these pleiotropic phenotypes, we see no evidence that they could have considerably affected our results. In the indicated text we consider different possible ways of how indirect effects of the LPS core mutants could have influenced our data, e.g., by changing the expression of bacterial surface proteins or by inducing the secretion of colanic acid (which is known to cause broad phage resistance). We argue that indirect effects through altered protein expression seem unlikely since the observed LPS core dependency correlates in no way with phage receptor specificity. In addition, *waaC* as well as *waaG* knockouts of *E. coli* K-12 were explicitly shown to not produce any relevant amount of colanic acid under conditions similar to our experimental setup.

Regarding TolC, our data show that only DanielBernoulli (Bas08) and no other tested phage (including all tested LPS-targeted phages) displayed reduced infectivity on the *tolC* knockout. This makes it difficult to imagine how pleiotropic LPS phenotypes of this mutant could have affected our results. Top agar plates showing the growth of several LPS-targeting phages on the *tolC* mutant are included as part of the complementation experiments (see above) in S9 Figure of the revised manuscript. Notably, DanielBernoulli is a close relative of several other known TolC-targeting phages and encodes the same *bona fide* RBP module (Fig 4B of the revised manuscript), making it highly plausible that the specific recognition of TolC by DanielBernoulli is true and no artifact. This aspect is now also discussed in the section “Considerations regarding the phenotyping of knockout mutants with pleiotropic outer membrane phenotypes” of the revised version of S2 Text.

*Genbank/INSD accession numbers are not yet available for the phages but these should be made available in the tables provided with the final submission.*

Reviewer #2 is right that no accession numbers for the phage genomes had been available upon submission. All genomes have in the meantime been uploaded to NCBI GenBank and accession numbers are now listed in the revised version of S5 Table. As is usual when describing new genomes, the sequences will be released by NCBI GenBank immediately upon publication of the manuscript.

*SPECIFIC COMMENTS*

*L 43: use eject instead of inject*

Done.

*L46: use virulent instead of lytic*

Done.

*L127: specify that these are phage isolates*

Done.

*L151: can you provide a citation for phages that use the ECA as a receptor?*

To the best of our knowledge, the use of ECA as *bona fide* phage receptor had only been shown for phage N4 before we also inferred it as the receptor of *Vequintavirinae* and relatives. The literature on the N4 example is cited, e.g., in lines 460f of the original submission. We considered whether we should highlight this specific example already in more detail at the early part of the manuscript as suggested by this reviewer, but then decided against it to avoid making this section already too detailed and complicated (which was a major concern of several reviewers).

*L185: not sure what the use of shortly is here. Breifly?*

Done.

*L227: these bona fide RBP loci*

Done.

*L329:* *this is imprecise language, the tail contraction may be described as syringe-like but the DNA ejection mechanism is presumably the same as for many sipho and podophages, driven by pressure differential.*

We adjusted the wording of this paragraph to remove the problematic ambiguity highlighted by reviewer #2: “Robust interaction with the primary receptor unpins the short tail fibers from the myovirus baseplate which enables their irreversible adsorption to terminal receptors in the LPS core. Consequently, the tail sheath contracts like a syringe and the phage tail penetrates the cell envelope to eject the viral genome [33] (Fig 7A).”

*L385-386: If ECA was the secondary receptor, would you not expect the change in phage sensitivity to be more drastic? The plating deficiency here seems to mostly be in the range of 1-2 log. Could loss of ECA have other effects on LPS structure that would account for the phenotype? Or is your model that ECA is dispensable?*

Reviewer #2 is right that the use of ECA as a secondary / terminal receptor should result in drastically reduced infectivity of these phages on the *wecB* mutant. However, we do not claim that the ECA is a secondary receptor of any bacteriophage in our study. For the *Vequintavirinae* that this reviewer is referring to (Fig 9 in the original submission, Fig 8 in the revised manuscript), we observe such a strong dependency only with LPS core mutants (Fig 9C old / Fig 8D new). This is in line with previous work highlighting the core LPS as secondary receptor of *Vequintavirinae* (discussed and referenced in lines 391-400 of the original submission). The “plating deficiency [...] in the range of 1-2 log” highlighted by this reviewer is indeed a strong indication that these phages use the ECA merely as a primary receptor. ECA binding would then greatly promote adsorption but not be absolutely essential for infection by most of these phages (at least on the *E. coli* K-12 strains with rough LPS that we used for phenotyping). In the absence of other evidence, we do not find it likely that the *wecB* knockout / loss of ECA has significant “other effects on LPS structure” because all tested LPS-dependent phages besides the *Vequintavirinae* do not show signs of impaired infectivity on the *wecB* mutant.

*L 419: ejection*

Done.

*Fig. 1: The podoviral model could also depict a central tail needle structure as observed in phage P22.*

We thank reviewer #2 for this suggestion, but Fig 1 has been removed from the revised manuscript in response to a comment of reviewer #1 (see above).

*Fig. 2: You may wish to label the boxes called Siphoviridae, Myovirdae and Podoviridae as "Other Siphovirdae", "Other Myoviridae" and "Other Podoviridae" as Sipho/Myo/Podo families are likely to be dissolved at some point and the genera they contain will be promoted to other families or sub-families.*

In this comment reviewer #2 suggests avoiding the currently valid families “*Siphoviridae*”, “*Myoviridae*”, and “*Podoviridae*” for phage classification because these families would likely be dissolved in the future. It is certainly right that bacteriophage taxonomy is a moving target and that these three families – historically containing all tailed phages – will at one point be replaced by a newer taxonomy. However, we do not feel that this is sufficient reason to not use the currently valid taxonomy of the ICTV (International Committee on Taxonomy of Viruses) as suggested by this reviewer. We therefore did not change these labels. Even if these highest-level categories are likely to be dissolved at one point, we feel that it is most intuitive to simply use the currently valid taxonomy and not some freely adjusted variant of it (also to avoid confusion of non-experts). In any case, the lower-level categories (subfamilies and genera) are likely to keep their names even if promoted to families / subfamilies so that there would be little room for true misunderstandings.

*All figures: Figure resolution is low, some of the graph axis labels are unreadable. Increase the text font sizes in the graphics where possible and be sure uo provide high-resolution figure in the final version.*

This is probably due to a conversion issue or another technical problem. We had uploaded the figures as high-resolution .tiff files that should have been available to reviewers (probably by clicking on the low-resolution preview in the peer review .pdf file). In any case, we also increased the size of graphical elements (and also axis labels etc.) on the figures in response to the comments of several reviewers. The revised versions of our figures should therefore be better readable and interpretable.

**Referees' comments – Reviewer #3**

*Systematic exploration of Escherichia coli phage-host interactions with the BASEL phage collection.*

*Maffei et al. report on a new valuable, well-characterized collection of coliphage isolated on a particular strain of Escherichia coli K-12. The characterization of the phage by sequence, parameters of infectivity, and interaction with specific phage defense systems is timely and well-done as is placing this information against ten-other well studied phage characterized in parallel. Providing a standard, systematic protocol for phage characterization and providing open access to the phage collection is a great service to the community. Overall, this is a strong paper making an important contribution to the coliphage biology field. We present our concerns and critical feedback below to improve the manuscript.*

*Broadly, this team isolates ~100 phages from diverse environmental sources, dissects the critical host molecular mechanisms governing each 66 phages using a collection of smartly chosen E. coli strains and goes so far as to assign possible (sometimes actual) phage molecular determinants as well. The systematic dissection of possible host surface receptors, interactions with E.coli restriction modification (RM) systems, - and abortive infection (Abi) systems for each phage class was elegant and well reasoned for the most part. While some caveats are probably justified due to expression of defense systems on plasmids, we don't think this really would modify the outcomes if done from the genome and indeed might lead to muddier conclusions. Comparison to basic infection on other E. coli and Salmonella strains was welcome.*

We thank reviewer #3 for this nuanced and constructive, generally positive comment on our work.

*This said, the isolation of these phage on K-12 strain is a possible limitation to the actual mechanistic diversity of the phage found despite the clear phylogenetic diversity of the collection. K-12 is an rfb mutant and thus has a rough phenotype with a complete LPS core and no O-antigen. It is known, for example, that many phage use the O-antigen as a receptor. That said, they did use a restored O16-antigen bearing K-12 to test their phage, as well as other related variants, at least addressing for their isolated phage what some of their interactions might be (as authors have alluded in S3 Text).*

Reviewer #3 is pointing at an important issue by highlighting the rough LPS phenotype of *E. coli* K-12 strains including the engineered derivative that we used as bait for phage sampling. As outlined also in considerable detail when replying to a similar point of reviewer #1 (see above), the use of hosts with rough LPS is not a specific limitation of our study because fundamental research on *E. coli* phages has so far almost exclusively used rough strains of the K-12 and B lineages (see Fig 12A of the original submission / Fig 11 of the revised manuscript).

Reviewer #3 is certainly right that “many phage use the O-antigen as a receptor” because for many phages recognition and binding of the O-antigen are likely necessary prerequisites of crossing the O-antigen barrier. However, the key question is whether these phages also *depend* on the O-antigen for infectivity (and would therefore be lost in our sampling strategy) or whether their O-antigen recognition is dispensable when cell surface receptors are directly accessible (i.e., in case of rough hosts like *E. coli* K-12). Whenever this has been studied, the O-antigen was mostly a merely optional first receptor like, e.g., in case of phage T5 and its relatives (nicely described by Golomidova et al., *Viruses* (2016); PMID 26805872). However, there are clearly also some examples like *Salmonella* phage P22 or *E. coli* phage G7C where O-antigen binding is essential (Broeker and Barbirz, *Mol Microbiol* (2017); PMID 28618013) - as reviewer #3 acknowledges, we have discussed this issue already at length in our original submission.

However, it had remained unknown so far which proportion of *E. coli* phages are dependent on host O‑antigen and would therefore be missing in our study. To address this question, we now performed quantitative phage isolation experiments using sewage inflow from four different treatment facilities and found that O-antigen-dependent *E. coli* phages are consistently very rare (few percent of isolates able to infect the host with O-antigen; see S6 Fig of the revised manuscript). These results are presented in the first section of the revised *Discussion* and more comprehensively in S2 Text of the revised manuscript (which was S3 Text in the original submission). Though it would be interesting to study the biology of these “O-antigen specialist” phages in more detail, we feel that their rarity and practical considerations (given that all used *E. coli* laboratory strains lack O-antigen) make their absence a rather minor limitation of the BASEL collection.

*Their dissection of the different ways the phage could evade the various restriction systems was elegant- largely delineated specifically encoded DNA protection functions and cut-site depletion strategies. Their discussion of trade-offs was however confusing. It wasn't clear their measurements demonstrated any real tradeoffs- just diversity. And the statement that these could be removed by engineering calls into question the idea that these are real tradeoffs in their minds. This we think remains to be tested.*

We thank reviewer #3 for this helpful comment regarding our discussion of possible phage trade-offs between phage traits. This concern was addressed in the revised manuscript by radically shortening the criticized subsection of the *Discussion* which removed all the indeed rather fuzzy statements criticized by reviewer #3. Throughout the manuscript we also weakened our claims regarding the detection of trade-offs and replaced them with formulations making it clearer that these are our interpretations. As an example, the revised abstract now reads more cautiously: “Our results also indicate strong trade-offs between fitness traits like broad host recognition and resistance to bacterial immunity that might drive the divergent adaptation of different phage groups to specific niches.”

In our view, the observed strengths and weaknesses of the different groups of phages are indeed indicative of trade-offs between phage traits as proposed previously (see the revised, shortened part of the *Discussion* now more cautiously entitled “Royal families instead of Darwinian Demon: The limits of bacteriophage evolution”). Our interpretation of the data fits well with the “royal family model” highlighted in this section as a possible explanation of how different groups of phages can stably coexist stably in space and time not only in marine environments (as originally proposed by Breitbart et al., *Nat Microbiol* (2010)) but also, as we observe together with previous work, in sewage and other habitats across the globe. Like reviewer #3, we look forward to future work that would unravel the molecular basis of the proposed trade-offs in more detail. An assorted, well-characterized set of phages like the BASEL collection would certainly be a helpful tool for this purpose.

*Authors do not provide support to their claims (line 31-33, 90-93 for example) that this work provides insights into "adaptation of different phage groups to specific niches" or that "These findings greatly expand our understanding of bacteriophage ecology, evolution, and their interplay with bacterial immunity systems". As mentioned earlier, these phages were isolated from diverse sources (though, mainly wastewater streams) on a parent lab strain of E. coli and the pattern of phage sensitivity to different restriction modification and Abi systems expressed in higher copy numbers do not explain the phage ecology, evolutionary pressures in natural environments and natural interplay of phages with all known and unknown bacterial immunity systems. Further, authors seem to claim that the phage infectivity pattern they see here determines the host range of phages (line 721), and we think this is a simplistic take on the complexity of phage host range. We question, is it possible to take these insights on possible phage receptors, sensitivity of phages to RM and Abi systems and predict the phage sensitivity of a non-model natural E coli strain variant. Addressing these questions and a systematic discussion on the utility of such a broadened phage collection would be highly valuable to highlight the importance of the work.*

*Finally, authors use a collection of E. coli K-12 single-gene (encoding surface protein) mutants ("KEIO collection") to determine probable receptors of BASEL phage collection and claim that this will serve as a powerful tool for future studies (line 33, 93, 722 for example). We question the generality of such an approach. Can we extend this characterization work to other model strains such as ESKAPE pathogens? What would make such an effort possible?*

*Obviously, it is known that there are multiple factors that mediate whether a particular mechanism can affect phage fitness on a host so even relatively close but wild strains of the same bacterium might not show the same susceptibilities or resistances to a model strain. It is a critical point that it has not been entirely proven how to generalize or formulate the results of a screen on a 'tame' collection of mutants like the KEIO collection to other strains much less to different classes of microbe. As raised earlier, the generality of the approach presented in this work itself is also difficult to argue. While it's accepted that the forward genetic methods are an effective method for discovery, they have proven hard to implement in diverse organisms. Even with advancement in newest functional genomic technologies that have come out recently, extending them to different pathogen classes and their natural population diversity such that this data can be effectively used for,*

*(as an example, design of phage therapies) is still, we think, in the distant future. Much of the data presented in this work (and other publications that have recently come out) point to a complex interaction among genetic factors (and environment) that mediate phage/host interactions and while there are certainly conserved themes in the specific modes of these mechanisms and their interactions currently prevent prediction of specific phage/host behaviors. We do believe however, that there may be systematic ways to explore host and phage diversity in nature such that a more generalizable predictive 'model' can be built. But it may require a much larger set of experiments than even this work puts forth.*

*We highly recommend addressing these points in the discussion to place the current work in the broader context and why would such an effort be valuable to the field in general.*

We thank reviewer #3 for these comments that we covered together because we feel that they are all linked and revolve around to important aspects:

1) Our work focuses on test tube and Petri dish experiments with phages infecting the *E. coli* K-12 laboratory strain which may or may not be informative about the behavior of different bacteriophages infecting, e.g., important pathogens of the ESKAPE group or even more difficult non-model organisms.

2) Our work reveals some links between genomic features and phenotypic behavior of bacteriophages but does not explore in any detail how far the knowledge of such links (e.g., with a lot more data) could be used to predict bacteriophage behavior in relevant contexts such as, e.g., when selecting phages for therapy.

We were limited in the space that we could devote to these comments in the main manuscript because they conflicted with the parallel goals of severely cutting down the text and simplifying its line of thoughts, especially since concerns similar to these here have not been raised by the other two reviewers. However, we agree with reviewer #3 that these are two important points and addressed them as follows:

ad 1): At different positions of our manuscript (first section of the *Results*, first section of the *Discussion*, and S2 Text) we argue in detail why we think that our BASEL collection is largely representative of the common groups of *E. coli* phages. Together with our systematic characterization, we see these phages as model organisms conceptually not unlike their *E. coli* K-12 host or, by extension, *Caenorhabditis elegans* worms, zebrafish, or mice. As we argue in our manuscript with view to the BASEL collection, such model organisms are invaluable for their potential to describe fundamental biological and evolutionary concepts which can then be validated in other organisms by targeted proof-of-concept studies (see, e.g., the article by J Rine, *Mol Biol Cell* (2014), PMID 24577733). Nevertheless, the use of model organisms is certainly inherently limited because not all findings can be equivalently transferred, e.g., from *E. coli* phages to those infecting other ESKAPE pathogens. It will be interesting to see future studies exploring, e.g., whether the taxonomic distribution of common phages infecting other hosts also fits the “royal family model” as we find for *E. coli* phages and as was originally described for marine communities by Breitbart et al., *Nat Microbiol* (2010). Similarly, the use of standard laboratory conditions like in almost all phage research of the last decades was an obvious choice to generate the systematic phenotypic data presented in our work, but is not without drawbacks. The real biology of *E. coli* and its phages does not happen in rich LB medium at constant aeration and 37°C, and future work could explore in more detail (maybe using the BASEL collection as a tool) how physiologically relevant changes of these conditions affect, e.g., the resistance and sensitivity of phages to immunity systems. Notably, it has long been suggested that testing the effective host range of bacteriophages under truly physiologically relevant conditions and not just under standard lab conditions might be an important prerequisite for reliable phage therapy: “[P] hage activity should ultimately be confirmed in the same environment in which the phage will be used for biocontrol of bacteria. Clearing a broth culture of bacteria is not equivalent to killing bacteria on the surface of an infected plant or animal [references]. Likewise, clearing a broth culture of planktonic bacteria is not equivalent to degrading a biofilm [references]” (P Hyman, *Pharmaceuticals* (2019), PMID 30862020). Our response to this first point is summarized in a new section entitled “Potential and limitations of the BASEL collection phages as new model organisms” in S2 Text of the revised manuscript.

ad 2) We agree with reviewer #3 that the field is currently far away from being able to predict phage-host interactions like the lysis or plaquing host range without direct experimentation because too many unknown parameters and complex interactions are involved. However, we feel that the BASEL collection is a step towards generating the kind of linked phenotypic and genomic data that would be required for such predictions. While complete host range predictions of newly isolated phages are far from reality even for *E. coli*, both previous work and our study show that such predictions are in principle possible at least for specific aspects of phage-host interactions. As an example, it has long been known (and broadly confirmed by our work, see Fig 7E of the revised manuscript) that the DNA hypermodification by T-even phages protects these viruses from most kinds of DNA-targeting immunity. This phenotype is therefore probably a safe prediction for all phages of this group if the presence of nucleotide modification pathways can be confirmed. A new discovery of our study was the clear relationship between the surface receptor specificity of diverse small siphoviruses and small loci encoding *bona fide* receptor-binding proteins that enabled us to predict the host receptor for basically all these phages infecting enterobacteria in the databases (Figs 4A and 4B as well as 3D, 5D, and 5E in the revised manuscript). A different yet conceptually related mechanism had previously been described for T5-like siphoviruses (*Markadamsvirinae*) and was likewise confirmed by our results (Figs 6C, 6D, and S3B in the revised manuscript). Other phenotypes would probably need a lot more data to achieve this degree of predictability. As an example, we find that the lateral tail fibers of the siphoviruses studied in our work are highly diverse and likely contact different variants of the ca. 200 types of *E. coli* O‑antigen (Figs S1C, S2A, and S3C). Due to the much higher number of different genetic variants a quantitatively small collection like our phages was insufficient to detect any systematic link between O‑antigen specificity and genetic variant of lateral tail fiber. However, we could also not experimentally test the O-antigen specificity of these phages besides the O16 O-antigen of K-12 (via the *wbbL(+)* variant of our *E. coli* K-12 host strain; see Fig 2A of the revised manuscript) and, indirectly, the different O-antigen types of the other enerobacterial strains that we used (see Figs 11 and 12 of the revised manuscript). We addressed this point by highlighting the value of different observed genotype / phenotype links throughout the manuscript (e.g., the examples highlighted above) and by expanding a question for future work that we raise at the end of the *Discussion* subsection entitled “Bacteriophage sensitivity and resistance to host immunity” of our revised manuscript.

*Minor Points:*

*Though we appreciate the effort in making these figures pretty, they are too crowded and make it difficult to read (at least in the pdf we have access to). Here are our suggestions*

*Fig 1, 2 and 3 can be combined into one, and Fig 1 and 2 need to be simplified. For example, we are not even sure Fig 1 is needed. Fig 2 has phage schematic representative of phages isolated. If they are TEM images that would have been highly valuable.*

Reviewer #3 is right that the twelve main manuscript figures of our original submission were too crowded which might make it difficult for some readers to extract the important information from them (reviewer #1 raised a similar point). We therefore removed Fig 1 from the manuscript (as implicitly suggested by reviewer #3). The design of figures 2 and 3 was slightly updated to increase clarity. More generally, the design of all figures was overhauled to declutter them and to increase clarity + accessibility. Briefly, for all figures showing genomic and phenotypic data of different groups of phages (Figs 3 + 5-10 in the revised manuscript / Figs 4 + 6-11 in the original submission) we introduced a consistent, simplified design and increased the size of many graphical elements. All more specific data were shifted to the supplemental figures.

Regarding TEM images: We feel that adding TEM micrographs of all 68 phage isolates is beyond the scope of our study because it would be a massive effort on its own just to produce these images of mostly illustrative value (but see our reply to the next concern of this reviewer).

*By the way, we thought having a TEM image of phages is one of the gold standard methods of characterization other than sequencing. We find not having TEM images associated with each phage surprising, and posit that those should be part of phage characterization workflows.*

We agree with reviewer #3 that TEM images are the gold standard when reporting totally novel phage isolates in papers that are focused on their first formal taxonomic description. However, all new isolates described in our manuscript belong to well-characterized phage groups for which the virion morphology has been extensively characterized. We therefore feel that TEM images of every single phage isolate would be beyond the scope of this work and would also not provide a lot of new information that could be integrated with the main body and focus of our study. Notably, the comments of reviewer #1 and reviewer #2 (who are also clearly phage experts) lack any reference to the absence of TEM micrographs.

However, reviewer #3 has a point that it would clearly be advantageous to include TEM micrographs in this manuscript. Most importantly, real TEM images instead of merely drawn cartoons of phages would certainly make our manuscript more accessible to non-experts. For this reason we have now included a TEM micrograph of a representative member of each group of phages that we sampled for the BASEL collection (see Figs 3 + 5-10 of the revised manuscript).

*In other figs, schematic of phage infection mode can be minimized by giving more space to the phenotyping data and phylogenetic trees for easy readability.*

The figures showing genomic and phenotypic data of different groups of phages (Figs 3 + 5-10 in the revised manuscript / Figs 4 + 6-11 in the original submission) were made more accessible by removing all more specific findings (to the supplemental material) and by increasing size and clarity of the core results shown in these figures (phylogenies and phenotypes). We also considered minimizing the schematics of phage infection but then decided to keep them in full size because they might be helpful for non-expert readers to understand the details of the phage infection process that are often at the core of arguments made in our manuscript.

*Fig 5 can be moved to Suppl info.*

Fig 5 of the original submission was kept because we feel that the surprising discovery of LptD as a rather common phage receptor is significant enough to deserve being shown in the main manuscript. However, in the revised manuscript we have now merged what had originally been Fig 5 with some of the general data on small siphovirus receptor specificity which had previously been placed non-intuitively in the illustration about *Drexlerviridae* phages (previously Fig 4, now Fig 3). We feel that this new figure (Fig 4 of the revised manuscript) serves as a good overview of all important aspects regarding small siphovirus receptor specificity. This topic is a major showcase of how combined genomic and phenotypic studies using the BASEL collection can generate significant new insight into bacteriophage biology and therefore, in our view, an important part of the manuscript.

*Fig 6 panel E can be moved to Suppl info.*

Done (now S2C Fig).

*The color coding of each phage name to a possible receptor needs to be mentioned in the legend.*

Done. We now also graphically highlight the color codes of phage names / receptors in every single figure.

*Fig 12B is complex and non legible, and needs to have higher image space, while LPS schematic can be reduced in size.*

Figure 12B of the original submission has been totally redesigned (now Figs 12A-H) to make it more accessible. We also included a small scheme (Fig 12A) that explains readers how to interpret the presentation of these data and that highlights how these data have been generated experimentally.

*As the RM and Abi systems are expressed from plasmids, how does different MOI of phage impact infection patterns? Did authors use different MOI to assess the sensitivity?*

Reviewer #3 is right that the multiplicity of infection (MOI) can influence the potency of bacterial immunity systems, e.g., in cases where multiple infections of the same host cell enable phages to shut off host immunity by dosage effects of phage-expressed genes (see, e.g., Borges et al., *Cell* (2018), PMID 30033364). We did not focus on this issue in our work but agree that it would be an excellent application of our BASEL collection to systematically study how the MOI affects the sensitivity / resistance of the various phage groups to the various immunity systems. Nevertheless, we implicitly tested a wide range of MOIs because our efficiency-of-plating (EOP) assays always challenged each host strain with a whole dilution series (eight logs) of phage stock (typically 10’10 pfu/ml; see *Materials and Methods* for details). In these assays, strong MOI effects would have been observed as an abrupt change in sensitivity or resistance between two phage dilutions because a given host (in the top agar) would be either sensitive or resistant to the phage depending on the local concentration of virions. We never saw any such effect. It is possible that, as reviewer #3 highlights, our practical choice to test (most) immunity systems cloned onto plasmids has affected our ability to detect these phenomena: Intuitively, a higher intracellular copy number of immunity system expression could protect the bacteria against simple dosage affects of high bacteriophage MOI. This would be an excellent field of future study but is, in our opinion, beyond the scope of our current work that already contains a huge amount of novel data on various other dimensions of phage-host interactions. We are discussing this issue as outlined here in a new, dedicated subsection of section “Considerations regarding the phenotyping of bacterial immunity systems” in S2 Text of the revised manuscript.

*Though authors have used single-gene mutants to assess the target receptor, gene complementation studies are not presented. If this work is setting up a standard of phage characterization, then authors need to clarify why not doing those experiments still support their claims similar to the point about TEM image made above.*

We agree with reviewer #3 that the complementation of single-gene mutants is the gold standard of genetic experiments to exclude, e.g., polar effects of the Keio collection kanamycin resistance cassette or suppressor mutations. In the revised manuscript we therefore show the complementation of every single mutant that we report to affect phage infection in our study. Briefly, we cloned a wildtype copy of the respective deleted or mutated gene of each mutant onto an SC101 *ori* plasmid (usually with its native promoter; see *Materials & Methods* of the revised manuscript) and repeated the key experiments with these strains. These data are now shown in supplemental figures S8 - S13 and demonstrate that every single mutant could be functionally complemented.

*Authors use "resistance" "sensitivity" "inhibition" "susceptibility" words to describe host and phage interaction. Clarifying their meaning in advance and keeping it consistent throughout would help readers. Further, clarifying the phenotypic data presented in Figs (to LPS mutants, RM and Abi systems) would be helpful. Specifically, clarifying the meaning of lower or higher EOP would be very helpful while going through the detailed text.*

Reviewer #3 is certainly right that a clear and consistent vocabulary is important for the accessibility of any manuscript to its readers. While streamlining and shortening the manuscript during the revision, we therefore took extra care to change and optimize ambiguous formulations that were sometimes also specifically highlighted by other reviewers (e.g., the list of “specific comments” of reviewer #2 and out replies). In addition, large part of our introduction and the first two sections of the results are devoted to explaining not only basic concepts but also basic terms of phage research and our methodology. This necessarily makes the text less succinct than, e.g., what reviewer #1 would have preferred (see above). However, we also do not like to expand it even further as implicitly suggested here by reviewer #3. In our view, the four words highlighted in this comment are commonly used in the biological sciences which should make them intuitive to the broad readership of PLoS Biology. We are not sure what reviewer #3 means when asking us to further “clarify[...] the phenotypic data presented in Figs”. Naturally, not every aspect of these data can be specifically highlighted and discussed commented in a manuscript that necessarily needs to focus on the biologically most important or interesting themes (although we highlight quite many aspects of these data throughout the text). We did not find instances where “lower EOP” or “higher EOP” (or similar formulations) were used in our manuscript.
